# Supplementary material for: A Search for Cyclin-Dependent Kinase 4/6 Inhibitors by Pharmacophore-Based Virtual Screening, Molecular Docking, and Molecular Dynamic Simulations
Source: Int J Mol Sci. 2021 Dec 14;22(24):13423. doi: 10.3390/ijms222413423 (PMC8706085; doi:10.3390/ijms222413423)
Supplement: Supplementary file 1 [file ijms-22-13423-s001.zip › ijms-1423218-supplementary.pdf]

SUPPLEMENTARY MATERIAL

A Search for Cyclin-Dependent Kinase 4/6 Inhibitors by Pharmacophore-Based Virtual Screening, Molecular Docking, and Molecular Dynamic Simulations

Ni Made Pitri Susanti [1,2], Sophi Damayanti [1], Rahmana Emran Kartasasmita[1] and Daryono Hadi Tjahjono [1,*]

[1] School of Pharmacy, Bandung Institute of Technology, Jalan Ganesha 10, Bandung 40132, Indonesia; dekpitsusanti@unud.ac.id (N.M.P.S); sophi.damayanti@fa.itb.ac.id (S.D); e.kartasasmita@gmail.com (R.E.K); daryonohadi@fa.itb.ac.id (D.H.T)

[2] Study Program of Pharmacy, Faculty of Mathematics and Natural Sciences, Universitas Udayana, Jalan Bukit Jimbaran, Badung, 80361, Indonesia

* Correspondence: daryonohadi@fa.itb.ac.id; Tel.: +62-812-2240-0120

Table S1. The internal validation results for pharmacophore model.

Pharmacophore		Validation Step									
Model	Feature	MOE2014					Pharmitt				
		GH	Se	Sp	Acc	Ya	GH	Se	Sp	Acc	Ya
1	F1, F2, F3	0.094	0.231	0.774	0.763	0.020	0.312	0.769	0.763	0.763	0.061
2	F1, F2, F4	0.396	1.000	0.828	0.831	0.104	0.346	0.846	0.748	0.750	0.063
3	F1, F2, F5	0.166	0.385	0.900	0.890	0.071	0.063	0.154	0.772	0.760	0.013
4	F1, F2, F6	0.497	1.000	0.571	0.579	0.045	0.604	1.000	0.458	0.469	0.036
5	F1, F2, F7	0.601	1.000	0.975	0.976	0.448	0.527	1.000	0.962	0.962	0.342
6	F1, F2, F8	0.406	1.000	0.755	0.760	0.076	0.412	0.923	0.652	0.658	0.050
7	F1, F2, F9	0.442	1.000	0.660	0.667	0.056	0.609	1.000	0.454	0.465	0.035
8	F1, F2, F10	0.406	1.000	0.882	0.884	0.144	0.342	0.846	0.766	0.768	0.067
9	F1, F3, F6	0.305	0.538	0.492	0.493	0.021	0.911	0.923	0.274	0.287	0.025
10	F1, F4, F6	0.552	1.000	0.506	0.516	0.039	0.626	0.923	0.405	0.415	0.030
11	F1, F5, F6	0.269	0.462	0.477	0.477	0.017	0.162	0.231	0.391	0.388	0.008
12	F1, F6, F7	0.837	1.000	0.325	0.338	0.029	0.817	0.923	0.306	0.318	0.026
13	F1, F6, F8	0.447	1.000	0.651	0.658	0.054	0.481	1.000	0.592	0.600	0.047
14	F1, F6, F9	0.570	1.000	0.488	0.498	0.038	0.683	1.000	0.402	0.413	0.032
15	F1, F6, F10	0.407	1.000	0.751	0.756	0.074	0.414	0.923	0.648	0.653	0.050
16	F1, F3, F7	0.093	0.231	0.806	0.795	0.023	0.288	0.692	0.728	0.727	0.048
17	F1, F4, F7	0.410	1.000	0.742	0.747	0.072	0.329	0.769	0.694	0.695	0.048
18	F1, F5, F7	0.278	0.692	0.783	0.781	0.060	0.246	0.538	0.634	0.632	0.029
19	F1, F7, F8	0.404	1.000	0.763	0.768	0.078	0.421	1.000	0.709	0.715	0.064
20	F1, F7, F9	0.415	1.000	0.726	0.732	0.068	0.235	0.538	0.675	0.673	0.032
21	F1, F7, F10	0.411	1.000	0.891	0.893	0.155	0.401	1.000	0.866	0.869	0.130
22	F2, F7, F9	0.403	1.000	0.766	0.771	0.079	0.419	1.000	0.714	0.719	0.065
23	F3, F7, F9	0.703	1.000	0.389	0.401	0.032	0.790	1.000	0.345	0.357	0.030

24	F4, F7, F9	0.472	1.000	0.606	0.614	0.048	0.561	0.923	0.455	0.465	0.033
25	F5, F7, F9	0.313	0.769	0.755	0.756	0.059	0.333	0.769	0.680	0.682	0.046
26	F6, F7, F9	0.442	1.000	0.660	0.667	0.056	0.449	1.000	0.646	0.653	0.053
27	F7, F9, F10	0.435	1.000	0.674	0.680	0.058	0.442	0.923	0.597	0.603	0.044
28	F1, F2, F3, F7	0.808	0.231	1.000	0.985	1.000	0.962	0.846	1.000	0.997	1.000
29	F1, F2, F4, F7	0.829	1.000	0.994	0.994	0.765	0.415	0.154	0.997	0.980	0.500
30	F1, F2, F5, F7	0.508	0.308	0.995	0.982	0.571	0.435	0.231	0.995	0.980	0.500
31	F1, F2, F6, F7	0.770	1.000	0.991	0.991	0.684	0.684	1.000	0.985	0.985	0.565
32	F1, F2, F7, F8	0.770	1.000	0.991	0.991	0.684	0.770	1.000	0.991	0.991	0.684
33	F1, F2, F7, F10	0.876	0.923	0.997	0.995	0.857	0.835	0.923	0.995	0.994	0.800
34	F1, F2, F3, F6	0.539	0.154	0.998	0.982	0.667	0.900	0.846	0.998	0.995	0.917
35	F1, F2, F4, F6	0.638	1.000	0.980	0.980	0.500	0.477	0.692	0.978	0.973	0.391
36	F1, F2, F5, F6	0.170	0.231	0.972	0.958	0.143	0.146	0.231	0.963	0.949	0.111
37	F1, F2, F6, F8	0.500	1.000	0.954	0.955	0.302	0.505	1.000	0.955	0.956	0.310
38	F1, F2, F6, F9	0.770	1.000	0.991	0.991	0.684	0.547	1.000	0.966	0.967	0.371
39	F1, F2, F6, F10	0.903	1.000	0.997	0.997	0.867	0.835	0.923	0.995	0.994	0.800
40	F1, F3, F6, F7	0.114	0.077	0.989	0.971	0.125	0.541	0.846	0.977	0.974	0.423
41	F1, F4, F6, F7	0.411	1.000	0.891	0.893	0.155	0.065	0.154	0.877	0.863	0.024
42	F1, F5, F6, F7	0.161	0.308	0.945	0.932	0.100	0.106	0.231	0.917	0.903	0.053
43	F1, F6, F7, F8	0.601	1.000	0.975	0.976	0.448	0.563	1.000	0.969	0.970	0.394
44	F1, F6, F7, F9	0.437	1.000	0.922	0.923	0.203	0.400	0.923	0.917	0.917	0.182
45	F1, F6, F7, F10	0.506	0.923	0.965	0.964	0.343	0.482	0.923	0.958	0.958	0.308
46	F1, F2, F7, F9	0.703	1.000	0.986	0.986	0.591	0.668	1.000	0.983	0.983	0.542
47	F2, F3, F7, F9	0.381	0.231	0.994	0.979	0.429	0.181	0.231	0.975	0.961	0.158
48	F2, F4, F7, F9	0.508	0.308	0.995	0.982	0.571	0.068	0.077	0.977	0.959	0.063

49	F2, F5, F7, F9	0.529	0.462	0.992	0.982	0.545	0.535	0.615	0.988	0.980	0.500
50	F2, F6, F7, F9	0.500	1.000	0.954	0.955	0.302	0.638	1.000	0.980	0.980	0.500
51	F2, F7, F8, F9	0.515	1.000	0.958	0.959	0.325	0.491	1.000	0.951	0.952	0.289
52	F2, F7, F9, F10	0.413	0.308	0.992	0.979	0.444	0.455	0.308	0.994	0.980	0.500
53	F2, F6, F7, F9	0.535	0.615	0.988	0.980	0.500	0.311	0.538	0.962	0.953	0.219
54	F3, F6, F7, F9	0.535	0.615	0.988	0.980	0.500	0.311	0.538	0.962	0.953	0.219
55	F4, F6, F7, F9	0.203	0.308	0.968	0.955	0.160	0.057	0.077	0.969	0.952	0.048
56	F5, F6, F7, F9	0.187	0.231	0.977	0.962	0.167	0.153	0.231	0.966	0.952	0.120
57	F6, F7, F8, F9	0.269	0.308	0.982	0.968	0.250	0.296	0.308	0.985	0.971	0.286
58	F6, F7, F9, F10	0.380	0.308	0.991	0.977	0.400	0.331	0.308	0.988	0.974	0.333
59	F1, F2, F6, F10	0.835	0.923	0.995	0.994	0.800	0.835	0.923	0.995	0.994	0.800
60	F1, F3, F6, F10	0.207	0.154	0.989	0.973	0.222	0.048	0.077	0.958	0.941	0.036
61	F1, F4, F6, F10	0.482	0.923	0.958	0.958	0.308	0.000	0.000	0.895	0.878	0.000
62	F1, F5, F6, F10	0.415	0.154	0.997	0.980	0.500	0.181	0.231	0.975	0.961	0.158
63	F1, F6, F7, F10	0.506	0.923	0.965	0.964	0.343	0.482	0.923	0.958	0.958	0.308
64	F1, F6, F8, F10	0.798	0.923	0.994	0.992	0.750	0.766	0.923	0.992	0.991	0.706
65	F1, F6, F9, F10	0.136	0.231	0.957	0.943	0.097	0.161	0.308	0.945	0.932	0.100
66	F1, F8, F9, F10	0.333	0.769	0.912	0.910	0.149	0.357	0.769	0.934	0.931	0.189
67	F2, F8, F9, F10	0.195	0.308	0.965	0.952	0.148	0.208	0.308	0.969	0.956	0.167
68	F3, F8, F9, F10	0.135	0.154	0.978	0.962	0.125	0.202	0.231	0.980	0.965	0.188
69	F4, F8, F9, F10	0.368	0.923	0.845	0.846	0.106	0.093	0.231	0.828	0.816	0.026
70	F5, F8, F9, F10	0.286	0.231	0.989	0.974	0.300	0.212	0.231	0.982	0.967	0.200
71	F6, F8, F9, F10	0.208	0.308	0.969	0.956	0.167	0.199	0.308	0.966	0.953	0.154
72	F7, F8, F9, F10	0.423	0.769	0.962	0.958	0.286	0.394	0.769	0.952	0.949	0.244
73	F1, F2, F3, F7, F10	0.788	0.154	1.000	0.983	1.000	0.769	0.077	1.000	0.982	1.000

74	F1, F2, F4, F7, F10	0.876	0.923	0.997	0.995	0.857	#DIV/0!	0.000	1.000	0.980	#DIV/0!
75	F1, F2, F5, F7, F10	0.788	0.154	1.000	0.983	1.000	0.788	0.154	1.000	0.983	1.000
76	F1, F2, F6, F7, F10	0.981	0.923	1.000	0.998	1.000	0.981	0.923	1.000	0.998	1.000
77	F1, F2, F7, F8, F10	0.981	0.923	1.000	0.998	1.000	0.981	0.923	1.000	0.998	1.000
78	F1, F2, F4, F7, F10	0.876	0.923	0.997	0.995	0.857	#DIV/0!	0.000	1.000	0.980	#DIV/0!
79	F1, F2, F3, F6, F10	0.788	0.154	1.000	0.983	1.000	0.769	0.077	1.000	0.982	1.000
80	F1, F2, F4, F6, F10	0.981	0.923	1.000	0.998	1.000	#DIV/0!	0.000	1.000	0.980	#DIV/0!
81	F1, F2, F5, F6, F10	0.788	0.154	1.000	0.983	1.000	0.788	0.154	1.000	0.983	1.000
82	F1, F2, F6, F8, F10	0.981	0.923	1.000	0.998	1.000	0.981	0.923	1.000	0.998	1.000
83	F1, F2, F6, F9, F10	0.808	0.231	1.000	0.985	1.000	0.827	0.308	1.000	0.986	1.000
84	F1, F2, F4, F6, F10	0.981	0.923	1.000	0.998	1.000	#DIV/0!	0.000	1.000	0.980	#DIV/0!
85	F1, F3, F6, F7, F10	0.788	0.154	1.000	0.983	1.000	0.769	0.077	1.000	0.982	1.000
86	F1, F4, F6, F7, F10	0.798	0.923	0.994	0.992	0.750	0.000	0.000	0.991	0.971	0.000
87	F1, F5, F6, F7, F10	0.808	0.231	1.000	0.985	1.000	0.539	0.154	0.998	0.982	0.667
88	F1, F6, F7, F8, F10	0.875	0.769	0.998	0.994	0.909	0.849	0.846	0.997	0.994	0.846
89	F1, F6, F7, F9, F10	0.508	0.308	0.995	0.982	0.571	0.455	0.308	0.994	0.980	0.500
90	F1, F4, F6, F7, F10	0.798	0.923	0.994	0.992	0.750	0.000	0.000	0.991	0.971	0.000

91	F1, F2, F7, F9, F10	0.827	0.308	1.000	0.986	1.000	0.827	0.308	1.000	0.986	1.000
92	F2, F3, F7, F9, F10	0.788	0.154	1.000	0.983	1.000	0.788	0.154	1.000	0.983	1.000
93	F2, F4, F7, F9, F10	0.827	0.308	1.000	0.986	1.000	0.769	0.077	1.000	0.982	1.000
94	F2, F5, F7, F9, F10	0.808	0.231	1.000	0.985	1.000	0.788	0.154	1.000	0.983	1.000
95	F2, F5, F8, F9, F10	0.788	0.154	1.000	0.983	1.000	0.808	0.231	1.000	0.985	1.000
96	F2, F6, F7, F9, F10	0.827	0.308	1.000	0.986	1.000	0.678	0.308	0.998	0.985	0.800
97	F2, F7, F8, F9, F10	0.827	0.308	1.000	0.986	1.000	0.827	0.308	1.000	0.986	1.000
98	F3, F5, F8, F9, F10	0.769	0.077	1.000	0.982	1.000	0.788	0.154	1.000	0.983	1.000
99	F3, F6, F7, F9, F10	0.808	0.231	1.000	0.985	1.000	0.788	0.154	1.000	0.983	1.000
100	F3, F7, F8, F9, F10	0.808	0.231	1.000	0.985	1.000	0.808	0.231	1.000	0.985	1.000
101	F4, F6, F7, F9, F10	0.827	0.308	1.000	0.986	1.000	0.395	0.077	0.998	0.980	0.500
102	F5, F6, F7, F9, F10	0.808	0.231	1.000	0.985	1.000	0.539	0.154	0.998	0.982	0.667
103	F5, F7, F8, F9, F10	0.788	0.154	1.000	0.983	1.000	0.788	0.154	1.000	0.983	1.000
104	F6, F7, F8, F9, F10	0.827	0.308	1.000	0.986	1.000	0.678	0.308	0.998	0.985	0.800
105	F1, F2, F3, F4, F7, F10	0.769	0.077	1.000	0.982	1.000	#DIV/0!	0.000	1.000	0.980	#DIV/0!
106	F1, F2, F3, F4, F5, F10	0.769	0.077	1.000	0.982	1.000	#DIV/0!	0.000	1.000	0.980	#DIV/0!
107	F1, F2, F3, F5, F6, F10	0.769	0.077	1.000	0.982	1.000	0.769	0.077	1.000	0.982	1.000

108	F1, F2, F3, F5, F7, F10	0.769	0.077	1.000	0.982	1.000	0.769	0.077	1.000	0.982	1.000
109	F1, F2, F3, F6, F7, F10	0.769	0.077	1.000	0.982	1.000	0.769	0.077	1.000	0.982	1.000
110	F1, F2, F4, F5, F6, F10	0.395	0.077	0.998	0.980	0.500	#DIV/0!	0.000	1.000	0.980	#DIV/0!
111	F1, F2, F4, F6, F7, F10	0.981	0.923	1.000	0.998	1.000	#DIV/0!	0.000	1.000	0.980	#DIV/0!
112	F1, F2, F5, F6, F8, F10	0.788	0.154	1.000	0.983	1.000	0.788	0.154	1.000	0.983	1.000
113	F1, F2, F5, F6, F9, F10	0.808	0.231	1.000	0.985	1.000	0.788	0.154	1.000	0.983	1.000
114	F1, F2, F5, F6, F7, F10	0.788	0.154	1.000	0.983	1.000	0.788	0.154	1.000	0.983	1.000
115	F1, F2, F5, F7, F8, F10	0.788	0.154	1.000	0.983	1.000	0.788	0.154	1.000	0.983	1.000
116	F1, F2, F5, F7, F9, F10	0.788	0.154	1.000	0.983	1.000	0.808	0.231	1.000	0.985	1.000
117	F1, F2, F6, F7, F8, F10	0.942	0.769	1.000	0.995	1.000	0.981	0.923	1.000	0.998	1.000
118	F1, F2, F6, F7, F9, F10	0.808	0.231	1.000	0.985	1.000	0.827	0.308	1.000	0.986	1.000
119	F1, F2, F7, F8, F9, F10	0.808	0.231	1.000	0.985	1.000	0.827	0.308	1.000	0.986	1.000
120	F1, F3, F4, F5, F7, F10	0.769	0.077	1.000	0.982	1.000	#DIV/0!	0.000	1.000	0.980	#DIV/0!
121	F1, F3, F4, F6, F7, F10	0.769	0.077	1.000	0.982	1.000	#DIV/0!	0.000	1.000	0.980	#DIV/0!
122	F1, F3, F5, F6, F7, F10	0.769	0.077	1.000	0.982	1.000	0.769	0.077	1.000	0.982	1.000
123	F1, F3, F6, F7, F8, F10	0.769	0.077	1.000	0.982	1.000	0.769	0.077	1.000	0.982	1.000
124	F1, F4, F5, F6, F7, F10	0.788	0.154	1.000	0.983	1.000	#DIV/0!	0.000	1.000	0.980	#DIV/0!

125	F1, F4, F5, F6, F8, F10	0.788	0.154	1.000	0.983	1.000	#DIV/0!	0.000	1.000	0.980	#DIV/0!
126	F1, F4, F6, F7, F8, F10	0.962	0.846	1.000	0.997	1.000	#DIV/0!	0.000	1.000	0.980	#DIV/0!
127	F1, F5, F6, F7, F8, F10	0.788	0.154	1.000	0.983	1.000	0.788	0.154	1.000	0.983	1.000
128	F1, F6, F7, F8, F9, F10	0.808	0.231	1.000	0.985	1.000	0.827	0.308	1.000	0.986	1.000
129	F2, F3, F5, F7, F9, F10	0.769	0.077	1.000	0.982	1.000	0.769	0.077	1.000	0.982	1.000
130	F2, F3, F5, F8, F9, F10	0.769	0.077	1.000	0.982	1.000	0.769	0.077	1.000	0.982	1.000
131	F2, F3, F6, F7, F9, F10	0.395	0.077	0.998	0.980	0.500	0.769	0.077	1.000	0.982	1.000
132	F2, F3, F6, F8, F9, F10	0.788	0.154	1.000	0.983	1.000	0.788	0.154	1.000	0.983	1.000
133	F2, F3, F7, F8, F9, F10	0.788	0.154	1.000	0.983	1.000	0.769	0.077	1.000	0.982	1.000
134	F2, F4, F5, F7, F9, F10	0.808	0.231	1.000	0.985	1.000	#DIV/0!	0.000	1.000	0.980	#DIV/0!
135	F2, F4, F5, F8, F9, F10	0.808	0.231	1.000	0.985	1.000	#DIV/0!	0.000	1.000	0.980	#DIV/0!
136	F2, F4, F7, F8, F9, F10	0.827	0.308	1.000	0.986	1.000	#DIV/0!	0.000	1.000	0.980	#DIV/0!
137	F2, F5, F6, F7, F9, F10	0.788	0.154	1.000	0.983	1.000	0.788	0.154	1.000	0.983	1.000
138	F3, F4, F5, F8, F9, F10	0.769	0.077	1.000	0.982	1.000	#DIV/0!	0.000	1.000	0.980	#DIV/0!
139	F3, F5, F6, F7, F9, F10	0.769	0.077	1.000	0.982	1.000	0.769	0.077	1.000	0.982	1.000
140	F3, F5, F7, F8, F9, F10	0.788	0.154	1.000	0.983	1.000	0.769	0.077	1.000	0.982	1.000
141	F4, F5, F6, F7, F8, F9	0.808	0.231	1.000	0.985	1.000	#DIV/0!	0.000	1.000	0.980	#DIV/0!

142	F4, F5, F6, F7, F9, F10	0.808	0.231	1.000	0.985	1.000	#DIV/0!	0.000	1.000	0.980	#DIV/0!
143	F4, F5, F7, F8, F9, F10	0.808	0.231	1.000	0.985	1.000	#DIV/0!	0.000	1.000	0.980	#DIV/0!
144	F4, F6, F7, F8, F9, F10	0.827	0.308	1.000	0.986	1.000	#DIV/0!	0.000	1.000	0.980	#DIV/0!
145	F5, F6, F7, F8, F9, F10	0.788	0.154	1.000	0.983	1.000	0.788	0.154	1.000	0.983	1.000
146	F1, F2, F3, F4, F5, F6, F7	0.769	0.077	1.000	0.982	1.000	#DIV/0!	0.000	1.000	0.980	#DIV/0!
147	F1, F2, F3, F5, F6, F7, F10	0.769	0.077	1.000	0.982	1.000	0.769	0.077	1.000	0.982	1.000
148	F1, F2, F4, F5, F6, F7, F10	0.788	0.154	1.000	0.983	1.000	#DIV/0!	0.000	1.000	0.980	#DIV/0!
149	F1, F2, F4, F5, F6, F7, F8	0.788	0.154	1.000	0.983	1.000	#DIV/0!	0.000	1.000	0.980	#DIV/0!
150	F1, F2, F4, F5, F6, F7, F9	0.808	0.231	1.000	0.985	1.000	#DIV/0!	0.000	1.000	0.980	#DIV/0!

F1: aromatic; F2: Aromatic; F3: Hydrophobic; F4; Hydrophobic, F5: Hydrophobic; F6: Hydrogen bond acceptor; F7: Hydrogen bond donor, F8: Hydrogen bond acceptor; F9: Hydrogen bond acceptor; F10: Hydrogen bond acceptor; GH: Güner-Henry; Se: Sensitivity; Sp: Specificity, Acc: Accuracy; Ya: Yield of active

Table S2. The independent validation results for pharmacophore model.

Pharmacophore		Validation Step									
Model	Feature	MOE2014					Pharmitt				
		GH	Se	Sp	Acc	Ya	GH	Se	Sp	Acc	Ya
1	F1, F2, F3	0.000	0.000	0.808	0.792	0.000	0.291	1.000	0.854	0.857	0.120
2	F1, F2, F4	0.289	1.000	0.852	0.855	0.119	0.202	0.800	0.794	0.794	0.072
3	F1, F2, F5	0.036	0.100	0.900	0.884	0.020	0.025	0.100	0.786	0.773	0.009
4	F1, F2, F6	0.160	1.000	0.564	0.573	0.044	0.147	1.000	0.524	0.533	0.040
5	F1, F2, F7	0.471	1.000	0.958	0.959	0.323	0.422	1.000	0.944	0.945	0.263
6	F1, F2, F8	0.240	1.000	0.772	0.776	0.081	0.205	1.000	0.692	0.698	0.061
7	F1, F2, F9	0.199	1.000	0.678	0.684	0.058	0.139	1.000	0.498	0.508	0.038
8	F1, F2, F10	0.277	0.900	0.872	0.873	0.123	0.202	0.900	0.738	0.741	0.064
9	F1, F3, F6	0.014	0.100	0.516	0.508	0.004	0.071	1.000	0.262	0.276	0.026
10	F1, F4, F6	0.118	1.000	0.428	0.439	0.034	0.092	1.000	0.338	0.351	0.029
11	F1, F5, F6	0.000	0.000	0.512	0.502	0.000	0.011	0.100	0.386	0.380	0.003
12	F1, F6, F7	0.087	1.000	0.322	0.335	0.029	0.083	1.000	0.306	0.320	0.028
13	F1, F6, F8	0.202	1.000	0.684	0.690	0.060	0.083	1.000	0.306	0.320	0.028
14	F1, F6, F9	0.114	1.000	0.416	0.427	0.033	0.092	1.000	0.338	0.351	0.029
15	F1, F6, F10	0.179	0.900	0.678	0.682	0.053	0.161	0.900	0.622	0.627	0.045
16	F1, F3, F7	0.000	0.000	0.848	0.831	0.000	0.268	1.000	0.822	0.825	0.101
17	F1, F4, F7	0.217	1.000	0.722	0.727	0.067	0.216	1.000	0.702	0.708	0.067
18	F1, F5, F7	0.143	0.500	0.842	0.835	0.060	0.105	0.500	0.704	0.700	0.033
19	F1, F7, F8	0.231	1.000	0.754	0.759	0.075	0.219	1.000	0.726	0.731	0.068
20	F1, F7, F9	0.201	1.000	0.700	0.706	0.059	0.191	1.000	0.656	0.663	0.055
21	F1, F7, F10	0.310	1.000	0.876	0.878	0.139	0.301	1.000	0.866	0.869	0.130
22	F2, F7, F9	0.220	1.000	0.730	0.735	0.069	0.338	1.000	0.900	0.902	0.167
23	F3, F7, F9	0.139	1.000	0.500	0.510	0.038	0.104	1.000	0.382	0.394	0.031

24	F4, F7, F9	0.155	1.000	0.550	0.559	0.043	0.094	1.000	0.344	0.357	0.030
25	F5, F7, F9	0.218	1.000	0.724	0.729	0.068	0.182	1.000	0.630	0.637	0.051
26	F6, F7, F9	0.201	1.000	0.682	0.688	0.059	0.199	1.000	0.678	0.684	0.058
27	F7, F9, F10	0.195	1.000	0.668	0.675	0.057	0.190	1.000	0.652	0.659	0.054
28	F1, F2, F3, F7	0.000	0.000	0.998	0.978	0.000	0.930	1.000	0.998	0.998	0.909
29	F1, F2, F4, F7	0.779	1.000	0.992	0.992	0.714	0.612	0.900	0.984	0.982	0.529
30	F1, F2, F5, F7	#DIV/0!	0.000	1.000	0.980	#DIV/0!	0.000	0.000	0.996	0.976	0.000
31	F1, F2, F6, F7	0.499	1.000	0.964	0.965	0.357	0.471	1.000	0.958	0.959	0.323
32	F1, F2, F7, F8	0.547	1.000	0.972	0.973	0.417	0.510	1.000	0.966	0.967	0.370
33	F1, F2, F7, F10	0.738	0.900	0.992	0.990	0.692	0.738	0.900	0.992	0.990	0.692
34	F1, F2, F3, F6	0.000	0.000	0.996	0.976	0.000	0.930	1.000	0.998	0.998	0.909
35	F1, F2, F4, F6	0.656	1.000	0.984	0.984	0.556	0.140	0.200	0.972	0.957	0.125
36	F1, F2, F5, F6	0.000	0.000	0.984	0.965	0.000	0.000	0.000	0.966	0.947	0.000
37	F1, F2, F6, F8	0.455	1.000	0.954	0.955	0.303	0.455	1.000	0.954	0.955	0.303
38	F1, F2, F6, F9	0.656	1.000	0.984	0.984	0.556	0.455	1.000	0.954	0.955	0.303
39	F1, F2, F6, F10	0.783	0.900	0.994	0.992	0.750	0.738	0.900	0.992	0.990	0.692
40	F1, F3, F6, F7	0.000	0.000	0.996	0.976	0.000	0.682	1.000	0.986	0.986	0.588
41	F1, F4, F6, F7	0.299	1.000	0.864	0.867	0.128	0.000	0.000	0.882	0.865	0.000
42	F1, F5, F6, F7	0.000	0.000	0.966	0.947	0.000	0.000	0.000	0.930	0.912	0.000
43	F1, F6, F7, F8	0.372	1.000	0.922	0.924	0.204	0.361	1.000	0.916	0.918	0.192
44	F1, F6, F7, F9	0.346	1.000	0.906	0.908	0.175	0.240	0.700	0.906	0.902	0.123
45	F1, F6, F7, F10	0.379	0.900	0.942	0.941	0.237	0.365	0.900	0.936	0.935	0.220
46	F1, F2, F7, F9	0.510	1.000	0.966	0.967	0.370	0.480	1.000	0.960	0.961	0.333
47	F2, F3, F7, F9	0.000	0.000	0.984	0.965	0.000	0.682	1.000	0.986	0.986	0.588
48	F2, F4, F7, F9	0.521	1.000	0.968	0.969	0.385	0.463	1.000	0.956	0.957	0.313

49	F2, F5, F7, F9	0.368	0.400	0.986	0.975	0.364	0.334	0.600	0.966	0.959	0.261
50	F2, F6, F7, F9	0.441	1.000	0.950	0.951	0.286	0.455	1.000	0.954	0.955	0.303
51	F2, F7, F8, F9	0.499	1.000	0.964	0.965	0.357	0.455	1.000	0.954	0.955	0.303
52	F2, F7, F9, F10	0.738	0.900	0.992	0.990	0.692	0.738	0.900	0.992	0.990	0.692
53	F2, F6, F7, F9	0.547	1.000	0.972	0.973	0.417	0.489	1.000	0.962	0.963	0.345
54	F3, F6, F7, F9	0.547	1.000	0.972	0.973	0.417	0.489	1.000	0.962	0.963	0.345
55	F4, F6, F7, F9	0.534	1.000	0.970	0.971	0.400	0.055	0.100	0.956	0.939	0.043
56	F5, F6, F7, F9	0.000	0.000	0.960	0.941	0.000	0.000	0.000	0.938	0.920	0.000
57	F6, F7, F8, F9	0.633	1.000	0.982	0.982	0.526	0.633	1.000	0.982	0.982	0.526
58	F6, F7, F9, F10	0.589	0.900	0.982	0.980	0.500	0.612	0.900	0.984	0.982	0.529
59	F1, F2, F6, F10	0.783	0.900	0.994	0.992	0.750	0.738	0.900	0.992	0.990	0.692
60	F1, F3, F6, F10	0.000	0.000	0.984	0.965	0.000	0.000	0.000	0.960	0.941	0.000
61	F1, F4, F6, F10	0.327	0.900	0.916	0.916	0.176	0.000	0.000	0.912	0.894	0.000
62	F1, F5, F6, F10	0.000	0.000	0.986	0.967	0.000	0.000	0.000	0.984	0.965	0.000
63	F1, F6, F7, F10	0.379	0.900	0.942	0.941	0.237	0.365	0.900	0.936	0.935	0.220
64	F1, F6, F8, F10	0.523	0.800	0.980	0.976	0.444	0.550	0.900	0.978	0.976	0.450
65	F1, F6, F9, F10	0.310	0.900	0.904	0.904	0.158	0.286	0.900	0.882	0.882	0.132
66	F1, F8, F9, F10	0.305	0.800	0.924	0.922	0.174	0.352	0.900	0.930	0.929	0.205
67	F2, F8, F9, F10	0.569	0.900	0.980	0.978	0.474	0.933	1.800	0.982	0.998	0.667
68	F3, F8, F9, F10	0.000	0.000	0.990	0.971	0.000	0.000	0.000	0.984	0.965	0.000
69	F4, F8, F9, F10	0.308	0.900	0.902	0.902	0.155	0.000	0.000	0.812	0.796	0.000
70	F5, F8, F9, F10	0.000	0.000	0.994	0.975	0.000	0.000	0.000	0.990	0.971	0.000
71	F6, F8, F9, F10	0.592	0.800	0.986	0.982	0.533	0.655	0.800	0.990	0.986	0.615
72	F7, F8, F9, F10	0.569	0.900	0.980	0.978	0.474	0.533	0.900	0.976	0.975	0.429
73	F1, F2, F3, F7, F10	#DIV/0!	0.000	1.000	0.980	#DIV/0!	#DIV/0!	0.000	1.000	0.980	#DIV/0!

74	F1, F2, F4, F7, F10	0.783	0.900	0.994	0.992	0.750	#DIV/0!	0.000	1.000	0.980	#DIV/0!
75	F1, F2, F5, F7, F10	#DIV/0!	0.000	1.000	0.980	#DIV/0!	#DIV/0!	0.000	1.000	0.980	#DIV/0!
76	F1, F2, F6, F7, F10	0.898	0.900	0.998	0.996	0.900	0.898	0.900	0.998	0.996	0.900
77	F1, F2, F7, F8, F10	0.975	0.900	1.000	0.998	1.000	0.975	0.900	1.000	0.998	1.000
78	F1, F2, F4, F7, F10	0.783	0.900	0.994	0.992	0.750	#DIV/0!	0.000	1.000	0.980	#DIV/0!
79	F1, F2, F3, F6, F10	#DIV/0!	0.000	1.000	0.980	#DIV/0!	#DIV/0!	0.000	1.000	0.980	#DIV/0!
80	F1, F2, F4, F6, F10	0.930	1.000	0.998	0.998	0.909	#DIV/0!	0.000	1.000	0.980	#DIV/0!
81	F1, F2, F5, F6, F10	#DIV/0!	0.000	1.000	0.980	#DIV/0!	#DIV/0!	0.000	1.000	0.980	#DIV/0!
82	F1, F2, F6, F8, F10	0.950	0.800	1.000	0.996	1.000	0.950	0.800	1.000	0.996	1.000
83	F1, F2, F6, F9, F10	0.950	0.800	1.000	0.996	1.000	0.930	1.000	0.998	0.998	0.909
84	F1, F2, F4, F6, F10	0.961	1.100	0.998	1.000	0.917	#DIV/0!	0.000	1.000	0.980	#DIV/0!
85	F1, F3, F6, F7, F10	#DIV/0!	0.000	1.000	0.980	#DIV/0!	#DIV/0!	0.000	1.000	0.980	#DIV/0!
86	F1, F4, F6, F7, F10	0.667	0.900	0.988	0.986	0.600	0.000	0.000	0.996	0.976	0.000
87	F1, F5, F6, F7, F10	0.000	0.000	0.994	0.975	0.000	0.000	0.000	0.994	0.975	0.000
88	F1, F6, F7, F8, F10	0.543	0.800	0.982	0.978	0.471	0.494	0.700	0.982	0.976	0.438
89	F1, F6, F7, F9, F10	0.741	0.800	0.994	0.990	0.727	0.741	0.800	0.994	0.990	0.727
90	F1, F4, F6, F7, F10	0.667	0.900	0.988	0.986	0.600	0.000	0.000	0.996	0.976	0.000

91	F1, F2, F7, F9, F10	0.898	0.900	0.998	0.996	0.900	0.821	1.200	0.990	0.994	0.706
92	F2, F3, F7, F9, F10	#DIV/0!	0.000	1.000	0.980	#DIV/0!	#DIV/0!	0.000	1.000	0.980	#DIV/0!
93	F2, F4, F7, F9, F10	0.939	1.200	0.996	1.000	0.857	0.000	0.000	0.996	0.976	0.000
94	F2, F5, F7, F9, F10	#DIV/0!	0.000	1.000	0.980	#DIV/0!	#DIV/0!	0.000	1.000	0.980	#DIV/0!
95	F2, F5, F8, F9, F10	#DIV/0!	0.000	1.000	0.980	#DIV/0!	#DIV/0!	0.000	1.000	0.980	#DIV/0!
96	F2, F6, F7, F9, F10	0.930	1.000	0.998	0.998	0.909	0.895	1.200	0.994	0.998	0.800
97	F2, F7, F8, F9, F10	#DIV/0!	0.900	1.000	0.998	#DIV/0!	0.906	1.100	0.996	0.998	0.846
98	F3, F5, F8, F9, F10	#DIV/0!	0.000	1.000	0.980	#DIV/0!	#DIV/0!	0.000	1.000	0.980	#DIV/0!
99	F3, F6, F7, F9, F10	#DIV/0!	0.000	1.000	0.980	#DIV/0!	#DIV/0!	0.000	1.000	0.980	#DIV/0!
100	F3, F7, F8, F9, F10	#DIV/0!	0.000	1.000	0.980	#DIV/0!	#DIV/0!	0.000	1.000	0.980	#DIV/0!
101	F4, F6, F7, F9, F10	0.898	0.900	0.998	0.996	0.900	0.000	0.000	0.998	0.978	0.000
102	F5, F6, F7, F9, F10	#DIV/0!	0.000	1.000	0.980	#DIV/0!	#DIV/0!	0.000	1.000	0.980	#DIV/0!
103	F5, F7, F8, F9, F10	#DIV/0!	0.000	1.000	0.980	#DIV/0!	0.000	0.000	0.998	0.978	0.000
104	F6, F7, F8, F9, F10	0.755	0.700	0.996	0.990	0.778	0.865	0.800	0.998	0.994	0.889
105	F1, F2, F3, F4, F7, F10	#DIV/0!	0.000	1.000	0.980	#DIV/0!	#DIV/0!	0.000	1.000	0.980	#DIV/0!
106	F1, F2, F3, F4, F5, F10	#DIV/0!	0.000	1.000	0.980	#DIV/0!	#DIV/0!	0.000	1.000	0.980	#DIV/0!
107	F1, F2, F3, F5, F6, F10	#DIV/0!	0.000	1.000	0.980	#DIV/0!	#DIV/0!	0.000	1.000	0.980	#DIV/0!

108	F1, F2, F3, F5, F7, F10	#DIV/0!	0.000	1.000	0.980	#DIV/0!	#DIV/0!	0.000	1.000	0.980	#DIV/0!
109	F1, F2, F3, F6, F7, F10	#DIV/0!	0.000	1.000	0.980	#DIV/0!	#DIV/0!	0.000	1.000	0.980	#DIV/0!
110	F1, F2, F4, F5, F6, F10	#DIV/0!	0.000	1.000	0.980	#DIV/0!	#DIV/0!	0.000	1.000	0.980	#DIV/0!
111	F1, F2, F4, F6, F7, F10	0.930	1.000	0.998	0.998	0.909	#DIV/0!	0.000	1.000	0.980	#DIV/0!
112	F1, F2, F5, F6, F8, F10	#DIV/0!	0.000	1.000	0.980	#DIV/0!	#DIV/0!	0.000	1.000	0.980	#DIV/0!
113	F1, F2, F5, F6, F9, F10	#DIV/0!	0.000	1.000	0.980	#DIV/0!	#DIV/0!	0.000	1.000	0.980	#DIV/0!
114	F1, F2, F5, F6, F7, F10	#DIV/0!	0.000	1.000	0.980	#DIV/0!	#DIV/0!	0.000	1.000	0.980	#DIV/0!
115	F1, F2, F5, F7, F8, F10	#DIV/0!	0.000	1.000	0.980	#DIV/0!	#DIV/0!	0.000	1.000	0.980	#DIV/0!
116	F1, F2, F5, F7, F9, F10	#DIV/0!	0.000	1.000	0.980	#DIV/0!	#DIV/0!	0.000	1.000	0.980	#DIV/0!
117	F1, F2, F6, F7, F8, F10	0.950	0.800	1.000	0.996	1.000	0.925	0.700	1.000	0.994	1.000
118	F1, F2, F6, F7, F9, F10	0.898	0.900	0.998	0.996	0.900	0.891	1.300	0.992	0.998	0.765
119	F1, F2, F7, F8, F9, F10	0.930	1.000	0.998	0.998	0.909	0.939	1.200	0.996	1.000	0.857
120	F1, F3, F4, F5, F7, F10	#DIV/0!	0.000	1.000	0.980	#DIV/0!	#DIV/0!	0.000	1.000	0.980	#DIV/0!
121	F1, F3, F4, F6, F7, F10	#DIV/0!	0.000	1.000	0.980	#DIV/0!	#DIV/0!	0.000	1.000	0.980	#DIV/0!
122	F1, F3, F5, F6, F7, F10	#DIV/0!	0.000	1.000	0.980	#DIV/0!	#DIV/0!	0.000	1.000	0.980	#DIV/0!
123	F1, F3, F6, F7, F8, F10	#DIV/0!	0.000	1.000	0.980	#DIV/0!	#DIV/0!	0.000	1.000	0.980	#DIV/0!
124	F1, F4, F5, F6, F7, F10	0.000	0.000	0.998	0.978	0.000	#DIV/0!	0.000	1.000	0.980	#DIV/0!

125	F1, F4, F5, F6, F8, F10	#DIV/0!	0.000	1.000	0.980	#DIV/0!	#DIV/0!	0.000	1.000	0.980	#DIV/0!
126	F1, F4, F6, F7, F8, F10	0.950	0.800	1.000	0.996	1.000	#DIV/0!	0.000	1.000	0.980	#DIV/0!
127	F1, F5, F6, F7, F8, F10	#DIV/0!	0.000	1.000	0.980	#DIV/0!	#DIV/0!	0.000	1.000	0.980	#DIV/0!
128	F1, F6, F7, F8, F9, F10	0.950	0.800	1.000	0.996	1.000	0.930	1.000	0.998	0.998	0.909
129	F2, F3, F5, F7, F9, F10	#DIV/0!	0.000	1.000	0.980	#DIV/0!	#DIV/0!	0.000	1.000	0.980	#DIV/0!
130	F2, F3, F5, F8, F9, F10	#DIV/0!	0.000	1.000	0.980	#DIV/0!	#DIV/0!	0.000	1.000	0.980	#DIV/0!
131	F2, F3, F6, F7, F9, F10	#DIV/0!	0.000	1.000	0.980	#DIV/0!	#DIV/0!	0.000	1.000	0.980	#DIV/0!
132	F2, F3, F6, F8, F9, F10	#DIV/0!	0.000	1.000	0.980	#DIV/0!	#DIV/0!	0.000	1.000	0.980	#DIV/0!
133	F2, F3, F7, F8, F9, F10	#DIV/0!	0.000	1.000	0.980	#DIV/0!	#DIV/0!	0.000	1.000	0.980	#DIV/0!
134	F2, F4, F5, F7, F9, F10	#DIV/0!	0.000	1.000	0.980	#DIV/0!	#DIV/0!	0.000	1.000	0.980	#DIV/0!
135	F2, F4, F5, F8, F9, F10	#DIV/0!	0.000	1.000	0.980	#DIV/0!	#DIV/0!	0.000	1.000	0.980	#DIV/0!
136	F2, F4, F7, F8, F9, F10	0.930	1.000	0.998	0.998	0.909	#DIV/0!	0.000	1.000	0.980	#DIV/0!
137	F2, F5, F6, F7, F9, F10	#DIV/0!	0.000	1.000	0.980	#DIV/0!	#DIV/0!	0.000	1.000	0.980	#DIV/0!
138	F3, F4, F5, F8, F9, F10	#DIV/0!	0.000	1.000	0.980	#DIV/0!	#DIV/0!	0.000	1.000	0.980	#DIV/0!
139	F3, F5, F6, F7, F9, F10	#DIV/0!	0.000	1.000	0.980	#DIV/0!	#DIV/0!	0.000	1.000	0.980	#DIV/0!
140	F3, F5, F7, F8, F9, F10	#DIV/0!	0.000	1.000	0.980	#DIV/0!	#DIV/0!	0.000	1.000	0.980	#DIV/0!
141	F4, F5, F6, F7, F8, F9	#DIV/0!	0.000	1.000	0.980	#DIV/0!	#DIV/0!	0.000	1.000	0.980	#DIV/0!

142	F4, F5, F6, F7, F9, F10	#DIV/0!	0.000	1.000	0.980	#DIV/0!	#DIV/0!	0.000	1.000	0.980	#DIV/0!
143	F4, F5, F7, F8, F9, F10	#DIV/0!	0.000	1.000	0.980	#DIV/0!	#DIV/0!	0.000	1.000	0.980	#DIV/0!
144	F4, F6, F7, F8, F9, F10	0.950	0.800	1.000	0.996	1.000	#DIV/0!	0.000	1.000	0.980	#DIV/0!
145	F5, F6, F7, F8, F9, F10	#DIV/0!	0.000	1.000	0.980	#DIV/0!	#DIV/0!	0.000	1.000	0.980	#DIV/0!
146	F1, F2, F3, F4, F5, F6, F7	#DIV/0!	0.000	1.000	0.980	#DIV/0!	#DIV/0!	0.000	1.000	0.980	#DIV/0!
147	F1, F2, F3, F5, F6, F7, F10	#DIV/0!	0.000	1.000	0.980	#DIV/0!	#DIV/0!	0.000	1.000	0.980	#DIV/0!
148	F1, F2, F4, F5, F6, F7, F10	#DIV/0!	0.000	1.000	0.980	#DIV/0!	#DIV/0!	0.000	1.000	0.980	#DIV/0!
149	F1, F2, F4, F5, F6, F7, F8	#DIV/0!	0.000	1.000	0.980	#DIV/0!	#DIV/0!	0.000	1.000	0.980	#DIV/0!
150	F1, F2, F4, F5, F6, F7, F9	#DIV/0!	0.000	1.000	0.980	#DIV/0!	#DIV/0!	0.000	1.000	0.980	#DIV/0!

F1: aromatic; F2: Aromatic; F3: Hydrophobic; F4; Hydrophobic, F5: Hydrophobic; F6: Hydrogen bond acceptor; F7: Hydrogen bond donor, F8: Hydrogen bond acceptor; F9: Hydrogen bond acceptor; F10: Hydrogen bond acceptor; GH: Güner-Henry; Se: Sensitivity; Sp: Specificity, Acc: Accuracy; Ya: Yield of active

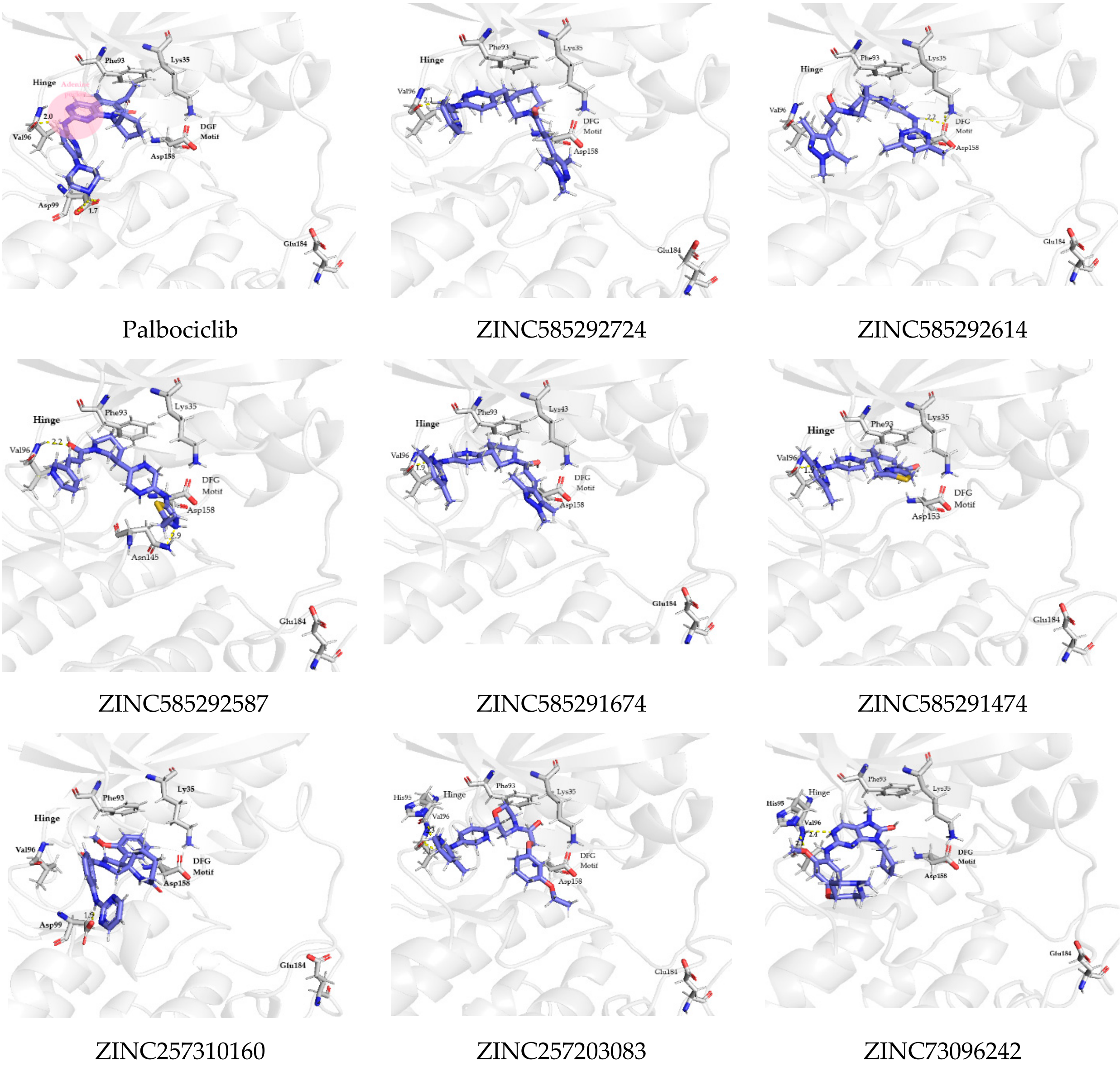

Figure S1. Ligand binding pose of palbociclib and hit compounds into the ATP-binding site of CDK4.

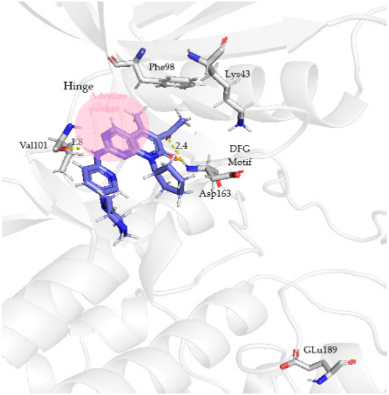

Palbociclib

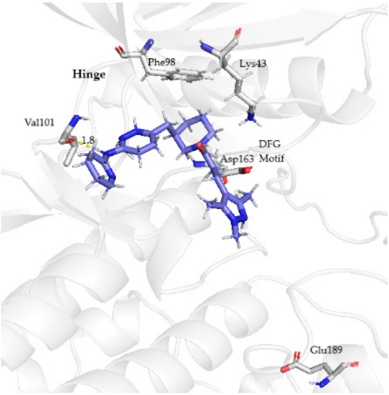

ZINC585292724

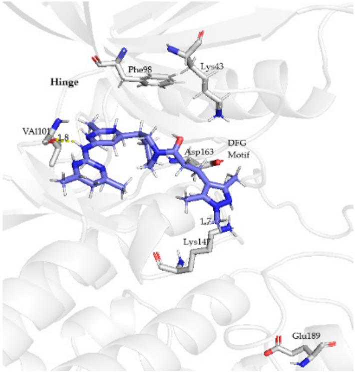

ZINC585292614

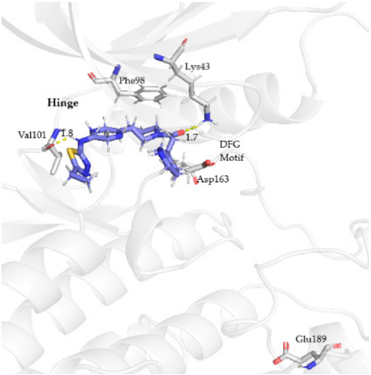

ZINC585292587

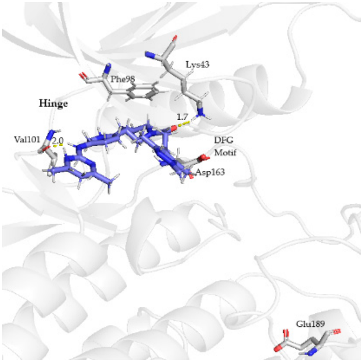

ZINC585291674

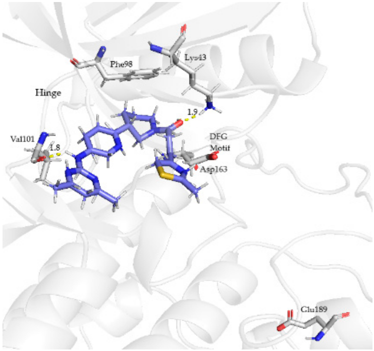

ZINC585291474

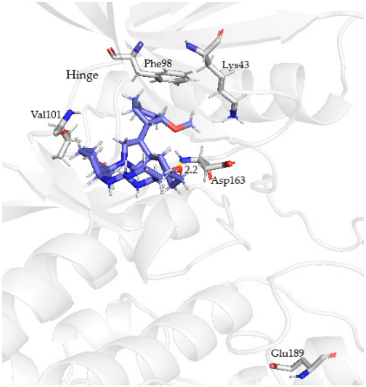

ZINC257310160

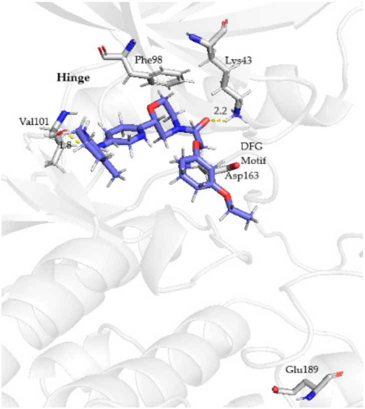

ZINC257203083

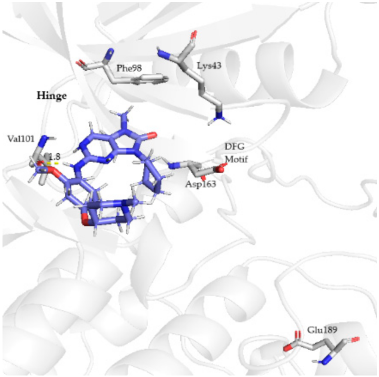

ZINC73096242

Figure S2. Ligand binding pose of palbociclib and hit compounds into the ATP-binding site of CDK6.

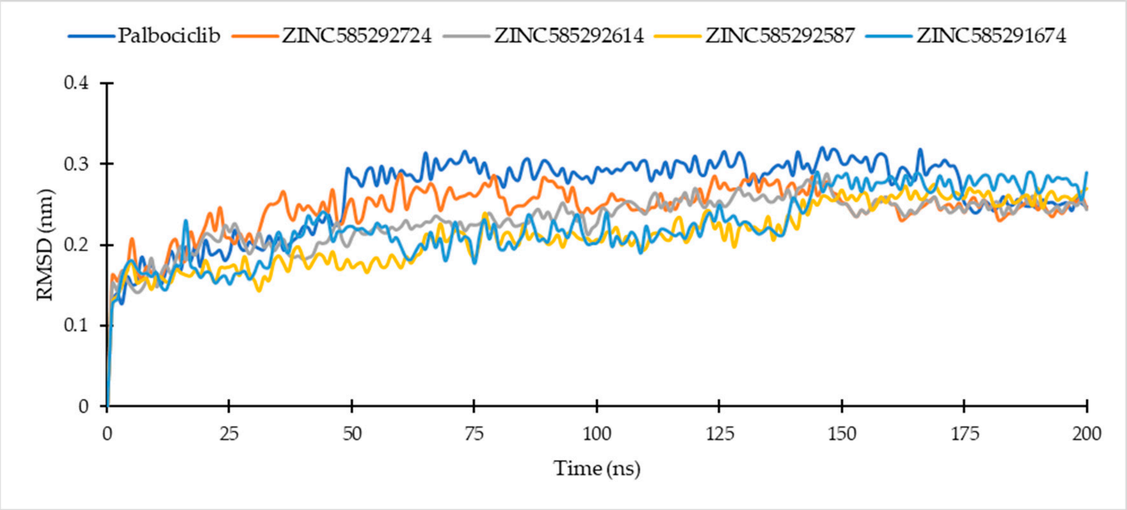

(a)

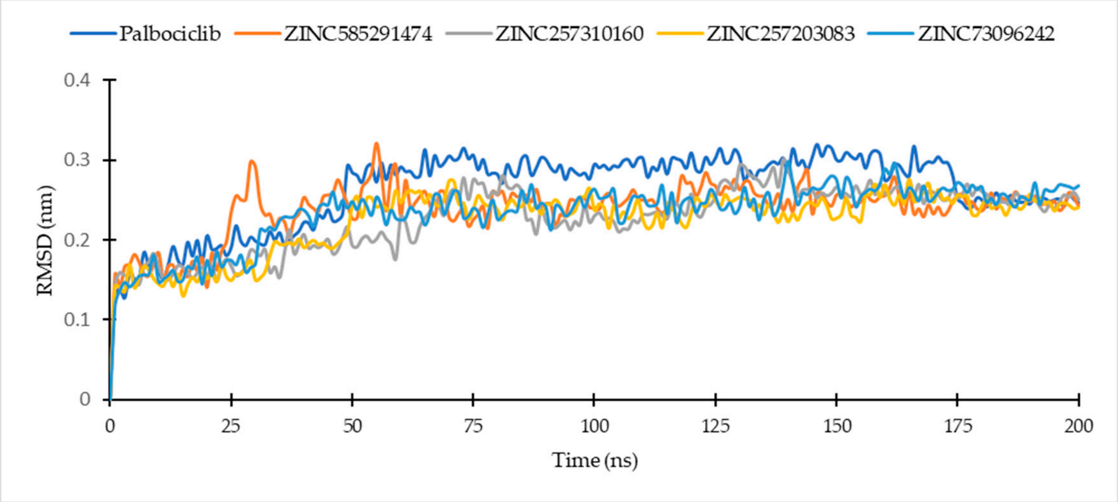

(b)

Figure S3: The backbone RMSD of the CDK4 complexes.

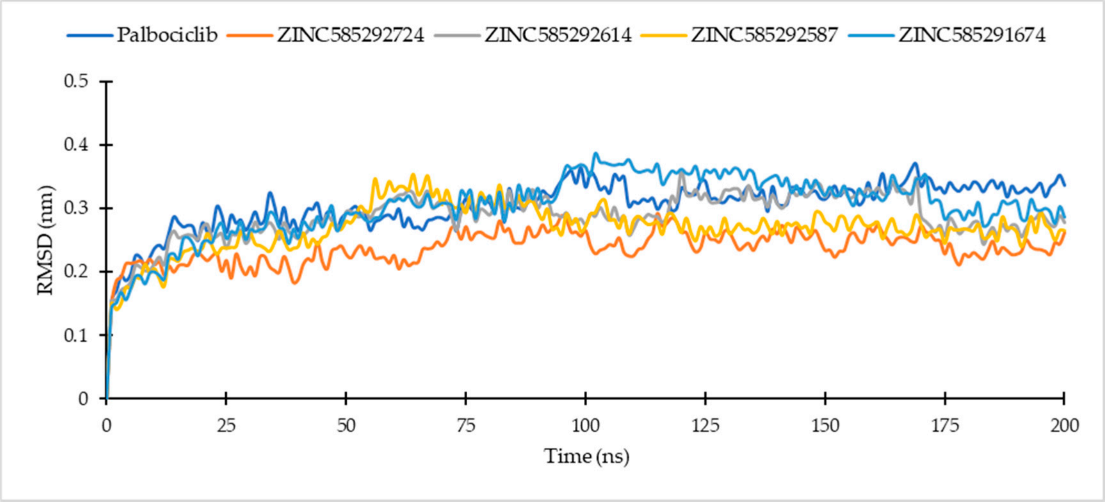

(a)

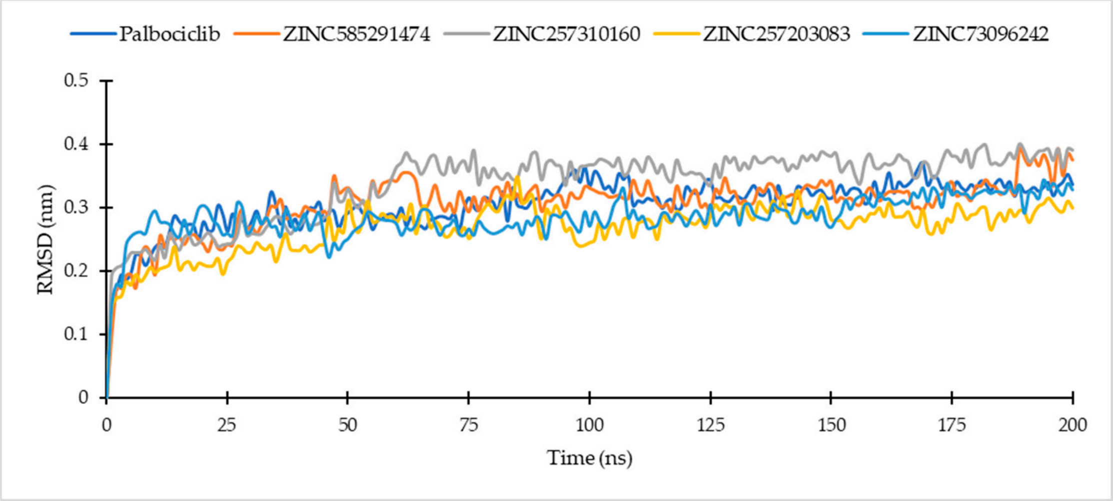

(b)

Figure S4: The backbone RMSD of the CDK6 complexes.

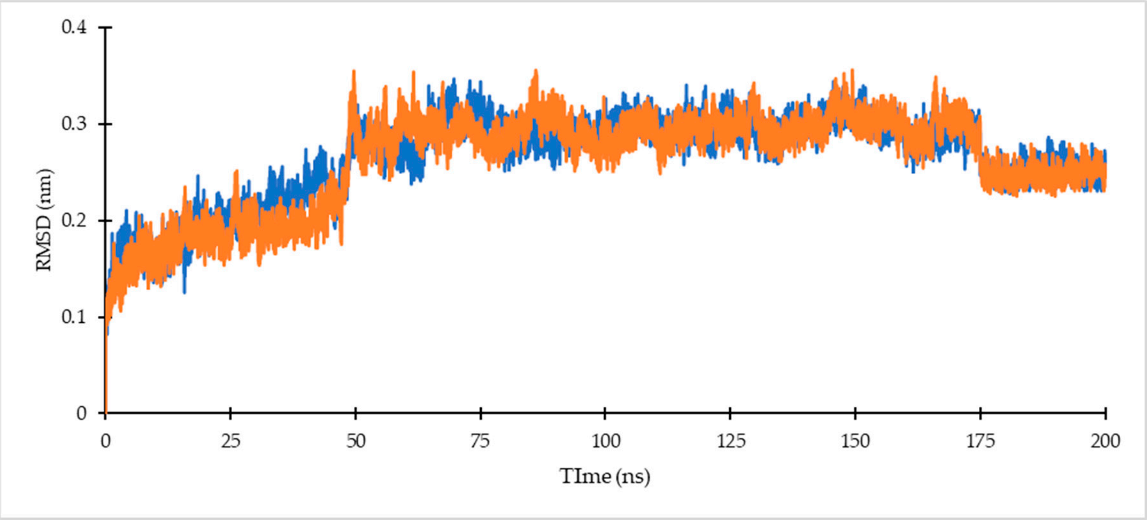

(a)

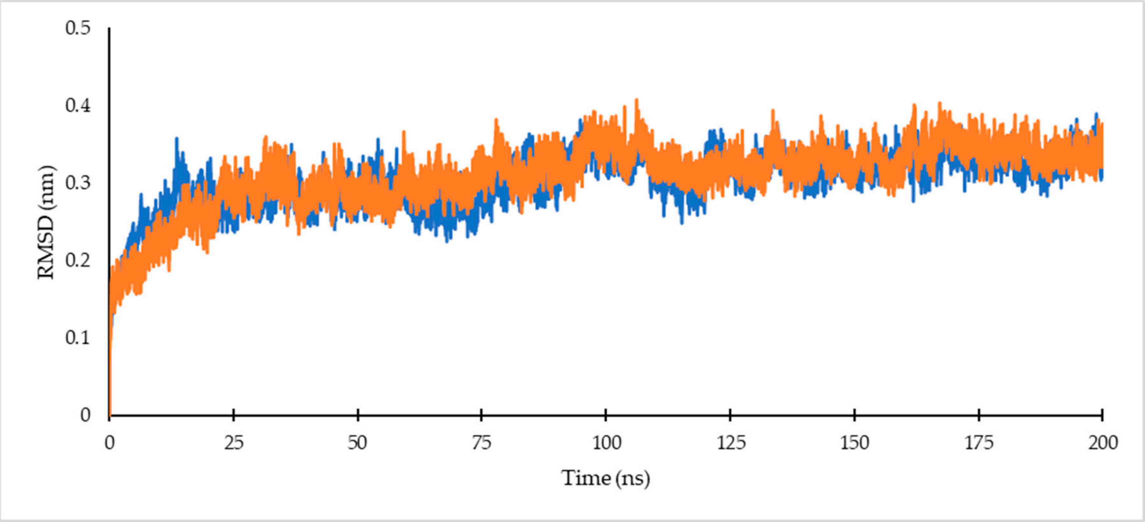

(b)

Figure S5. The backbone RMSD of the CDK4 (a) and CDK6 (b) in complexes with Palbociclib. Color code: Blue: Replica 1; Orange: Replica 2.

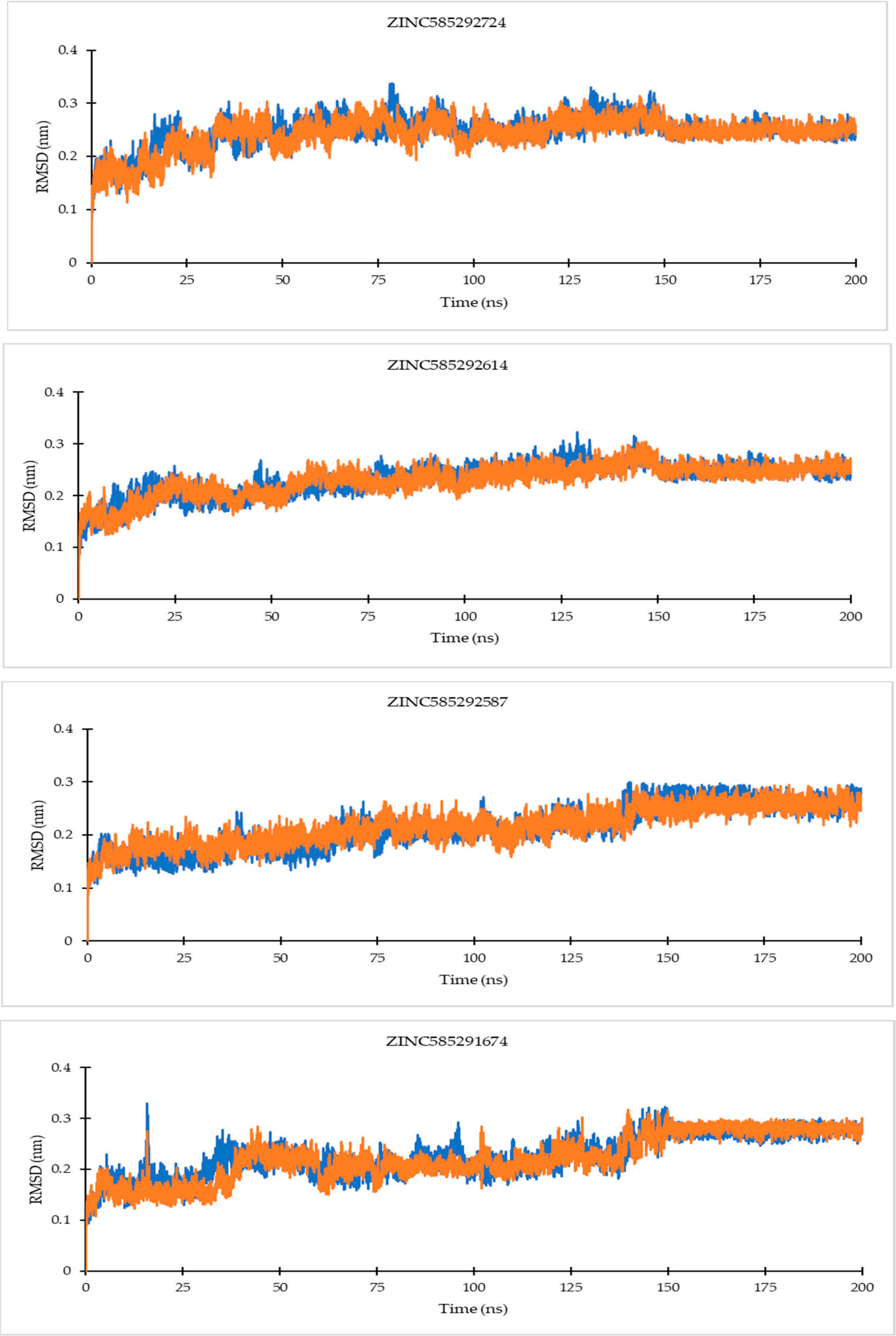

Figure S6. The backbone RMSD of the CDK4 complexes with ZINC585292724, ZINC585292614, ZINC585292587, and ZINC585291674. Color code: Blue: Replica 1; Orange: Replica 2.

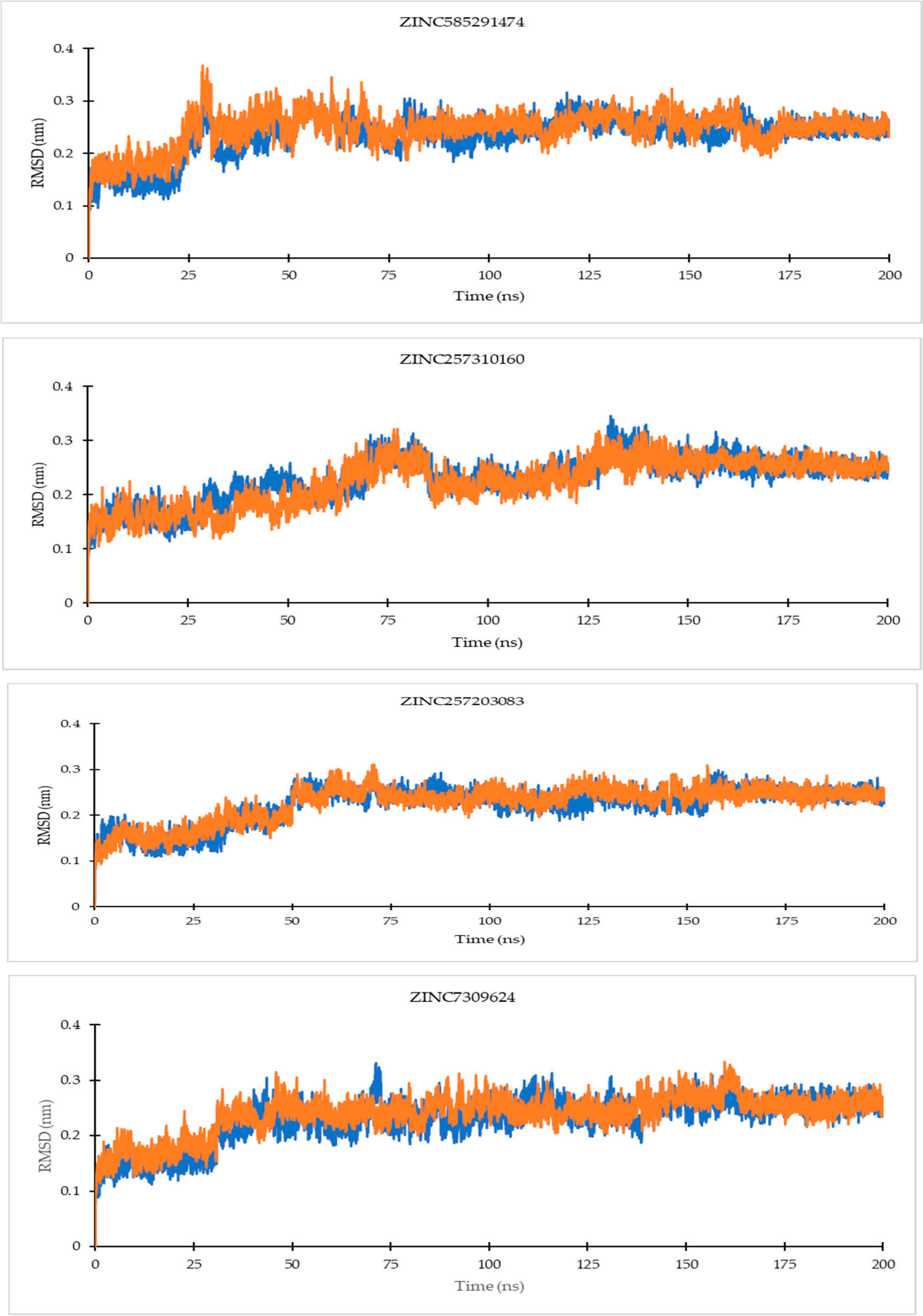

Figure S7. The backbone RMSD of the CDK4 complexes with ZINC585291474, ZINC257310160, ZINC257203083, and ZINC73096242. Color code: Blue: Replica 1; Orange: Replica 2.

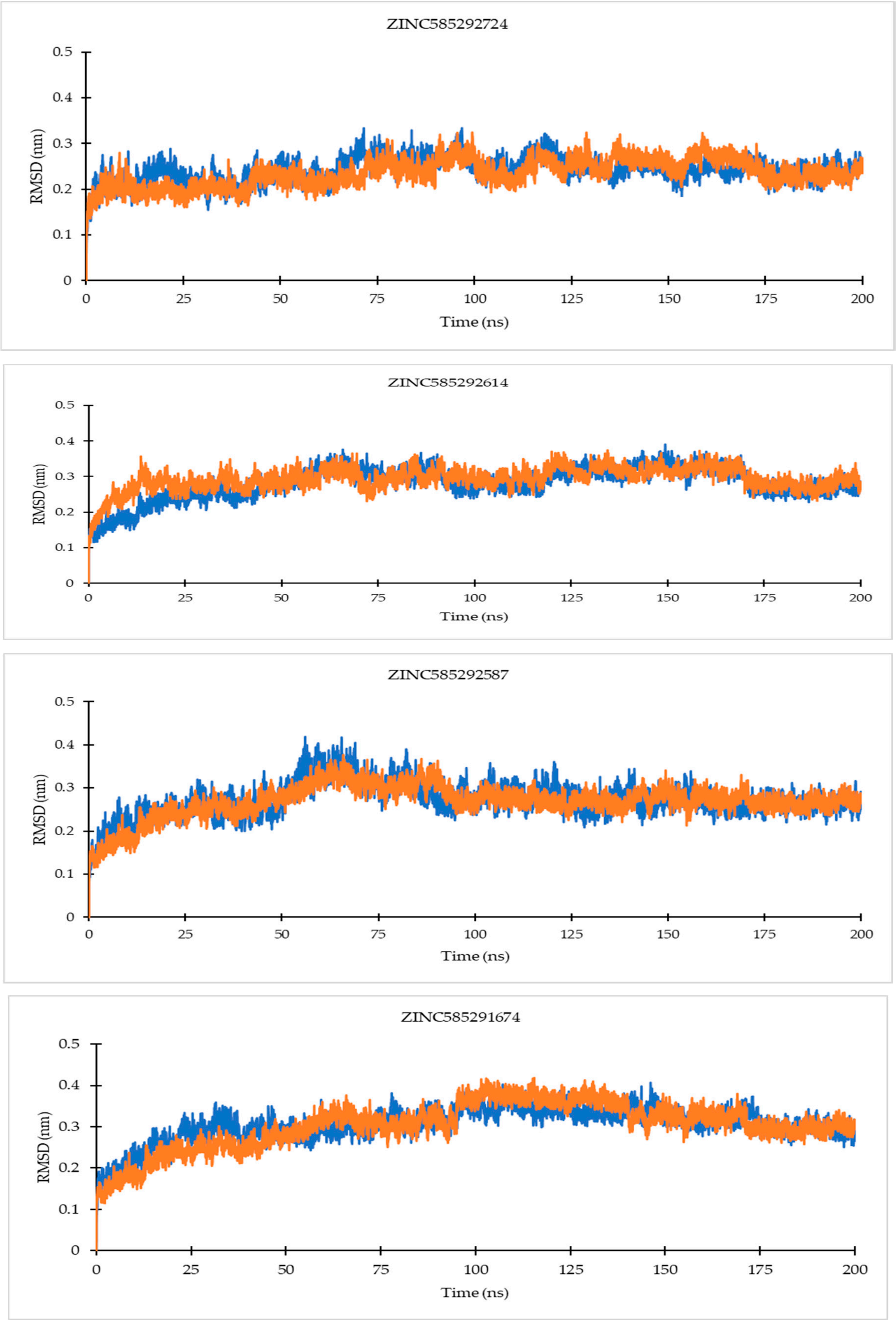

Figure S8: The backbone RMSD of the CDK6 complexes with ZINC585292724, ZINC585292614, ZINC585292587, and ZINC585291674. Color code: Blue: Replica 1; Orange: Replica 2.

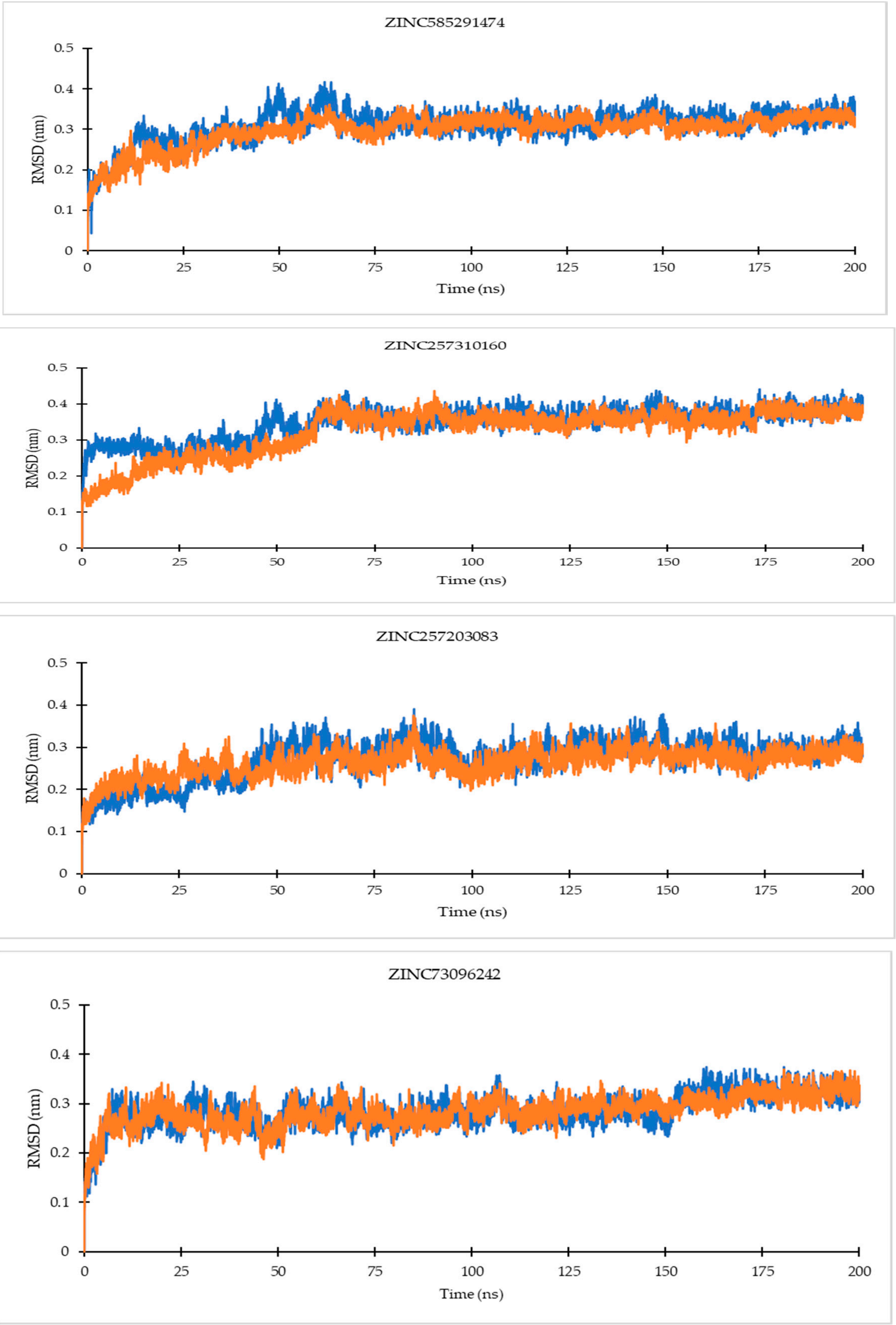

Figure S9: The backbone RMSD of the CDK6 complexes with ZINC585291474, ZINC257310160, ZINC257203083, and ZINC73096242. Color code: Blue: Replica 1; Orange: Replica 2.

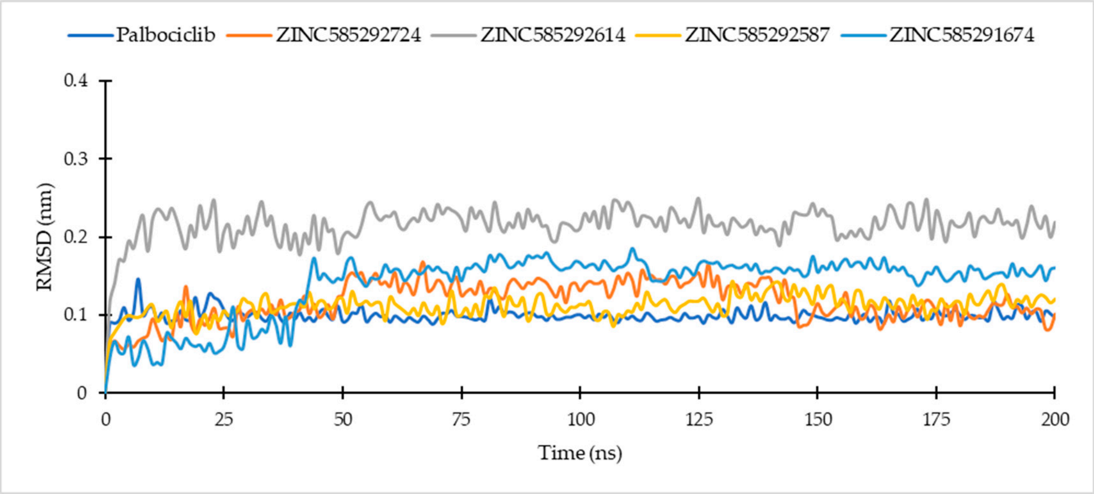

(a)

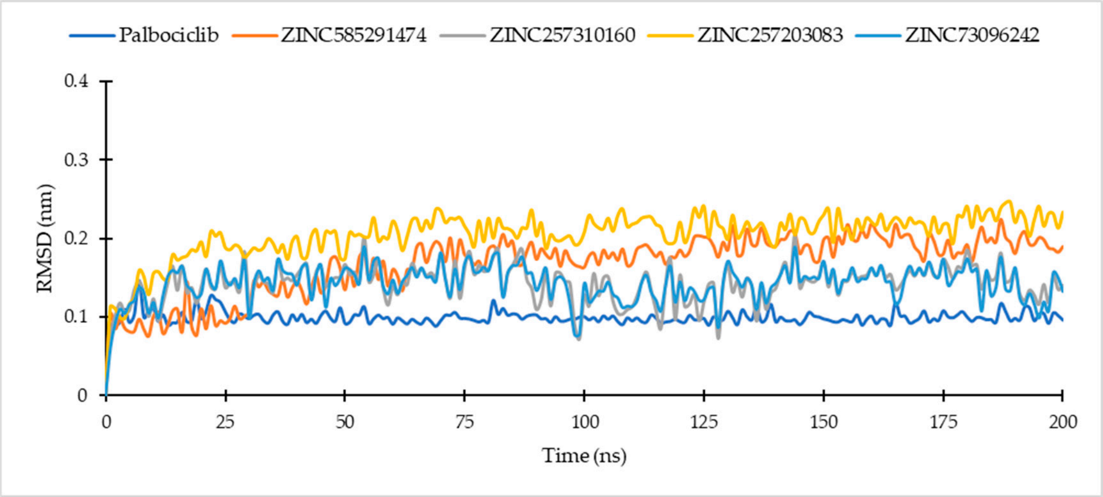

(b)

Figure S10: The ligand RMSD of the CDK4 complexes.

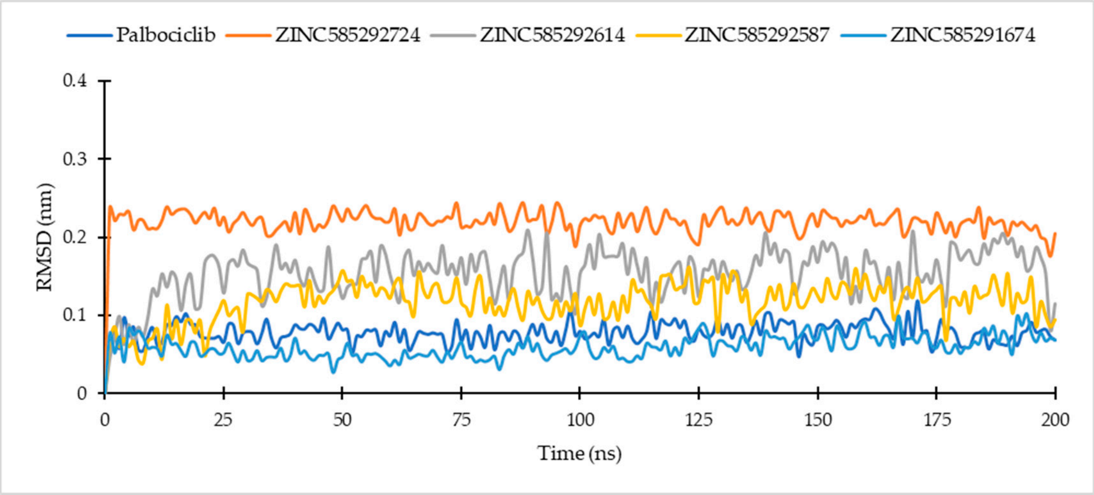

(a)

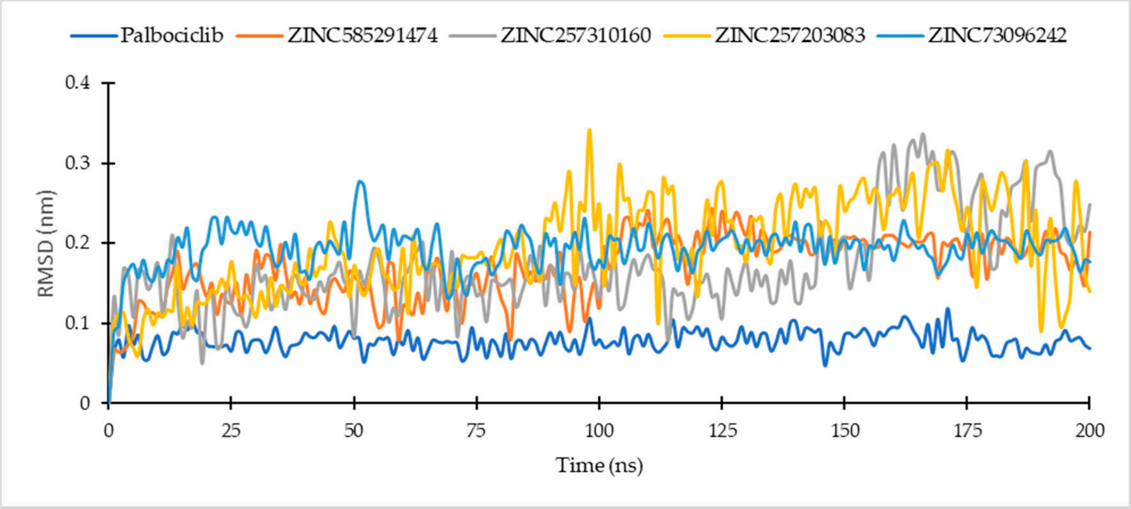

(b)

Figure S11: The ligand RMSD of the CDK6 complexes.

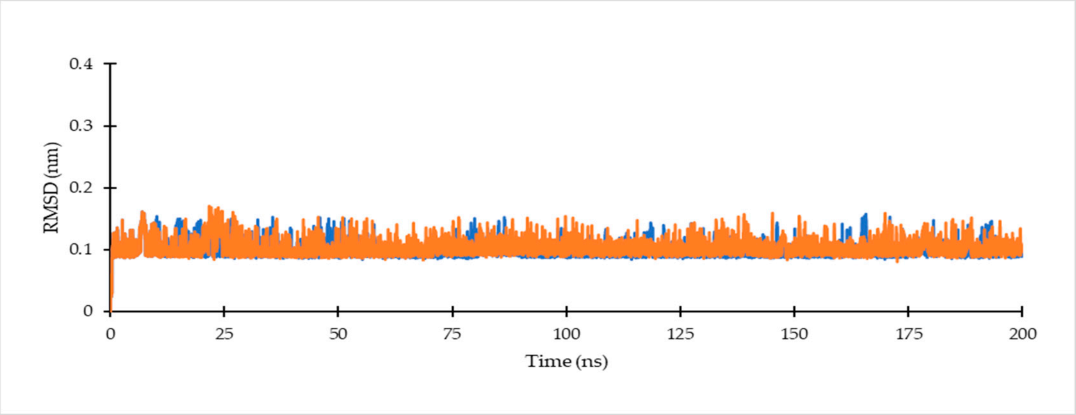

(a)

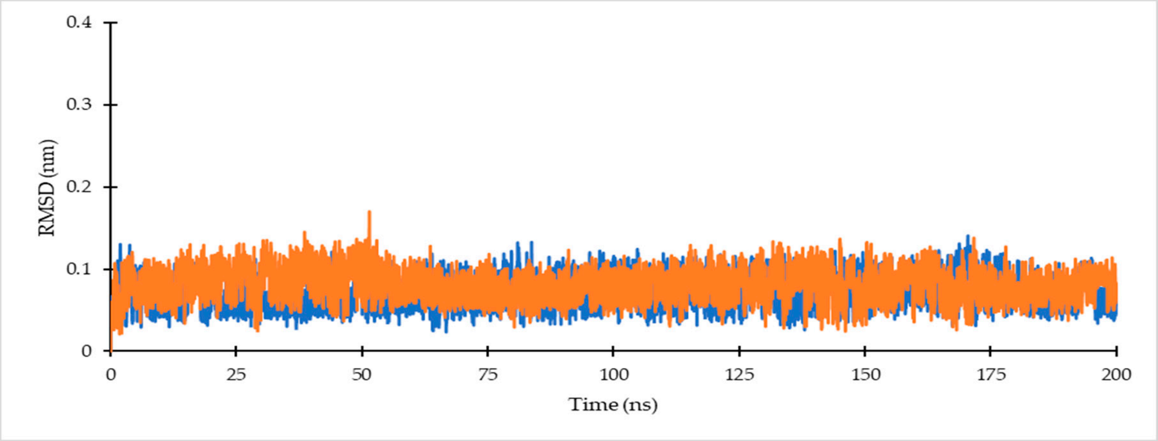

(b)

Figure S12. The Ligand RMSD of the CDK4 (a) and CDK6 (b) complexes with Palbociclib. Color code: Blue: Replica 1; Orange: Replica 2.

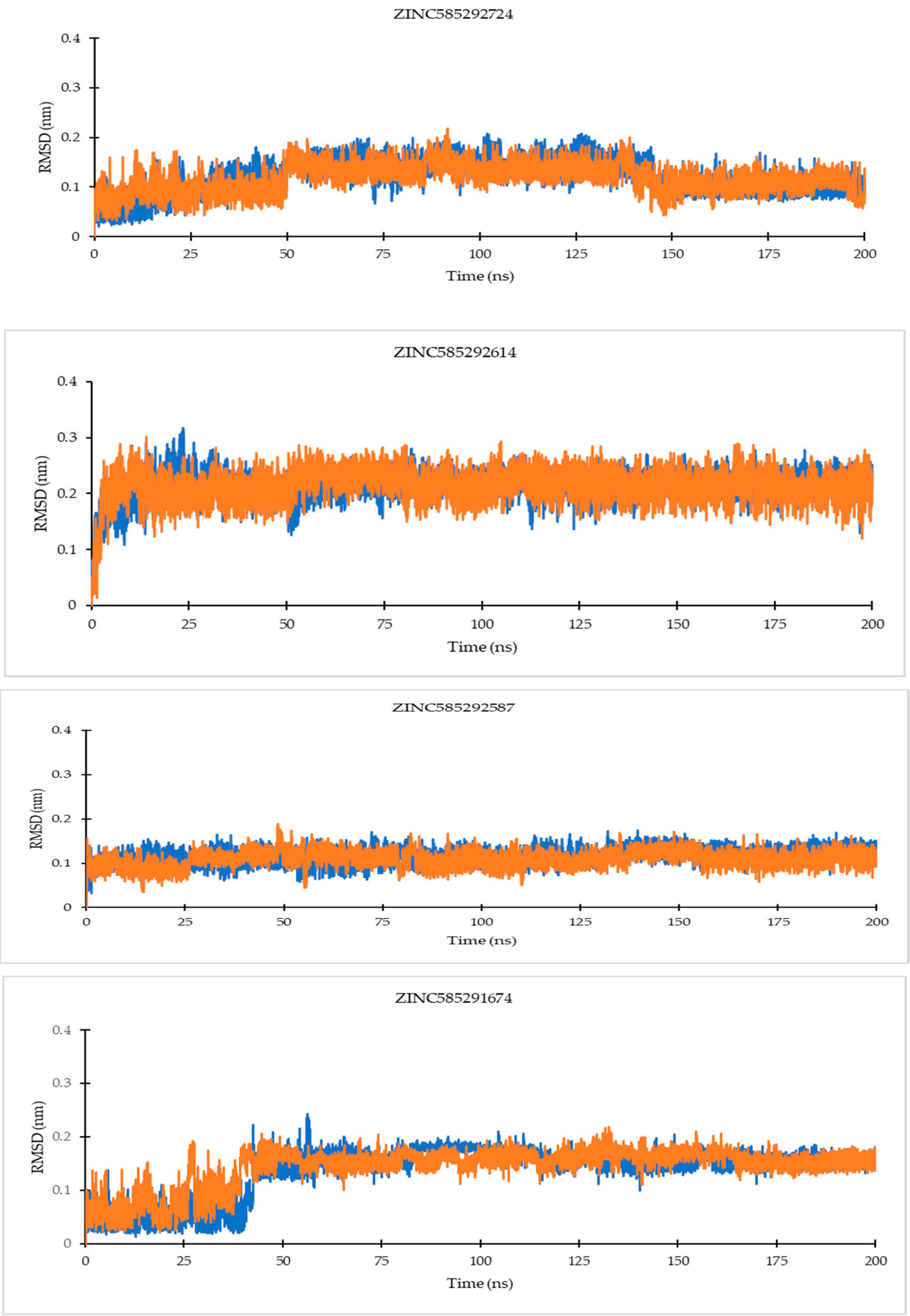

Figure S13. The ligand RMSD of the CDK4 complexes with ZINC585292724, ZINC585292614, ZINC585292587, and ZINC585291674. Color code: Blue: Replica 1; Orange: Replica 2.

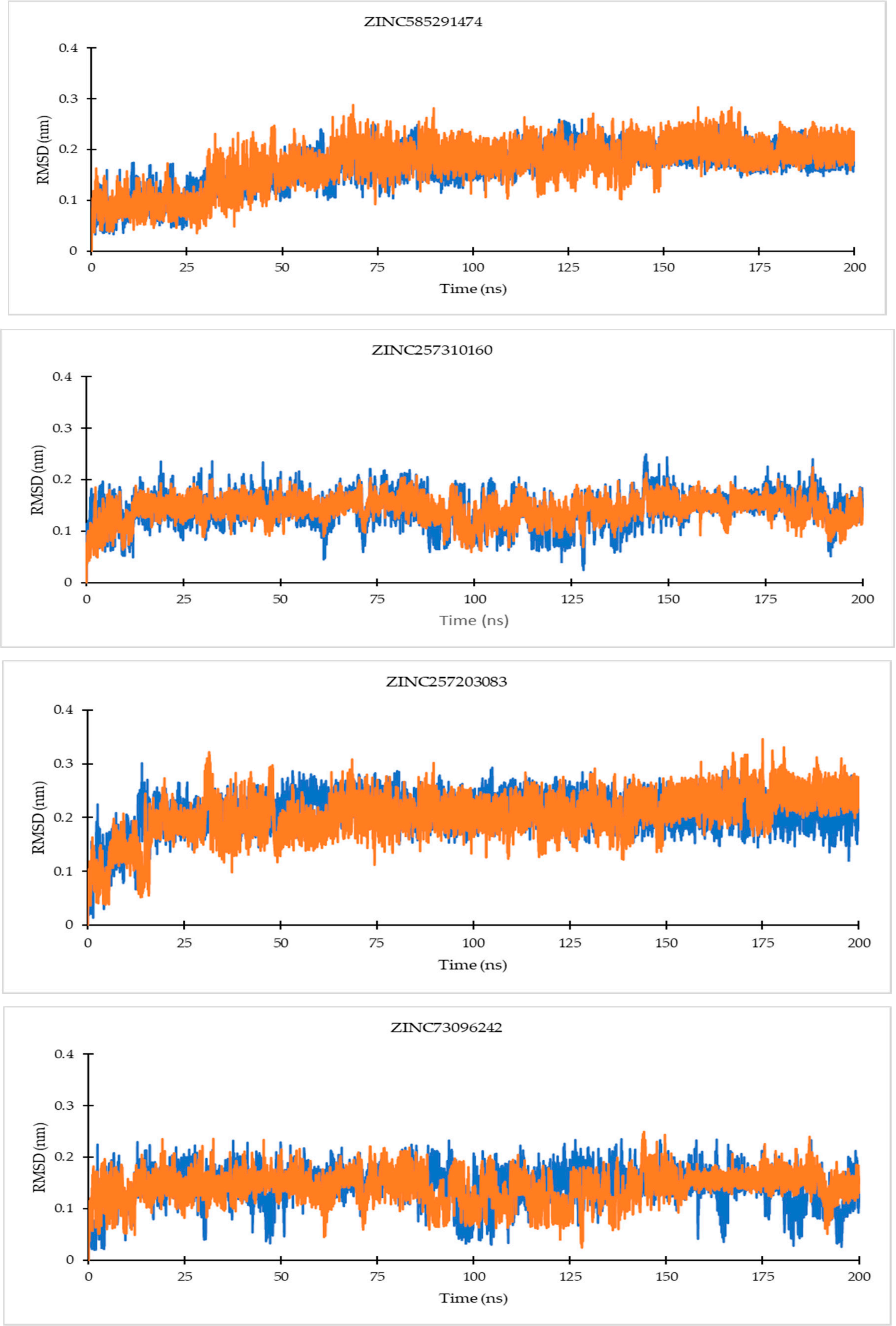

Figure S14. The ligand RMSD of the CDK4 complexes with ZINC585291474, ZINC257310160, ZINC257203083, and ZINC73096242. Color code: Blue: Replica 1; Orange: Replica 2.

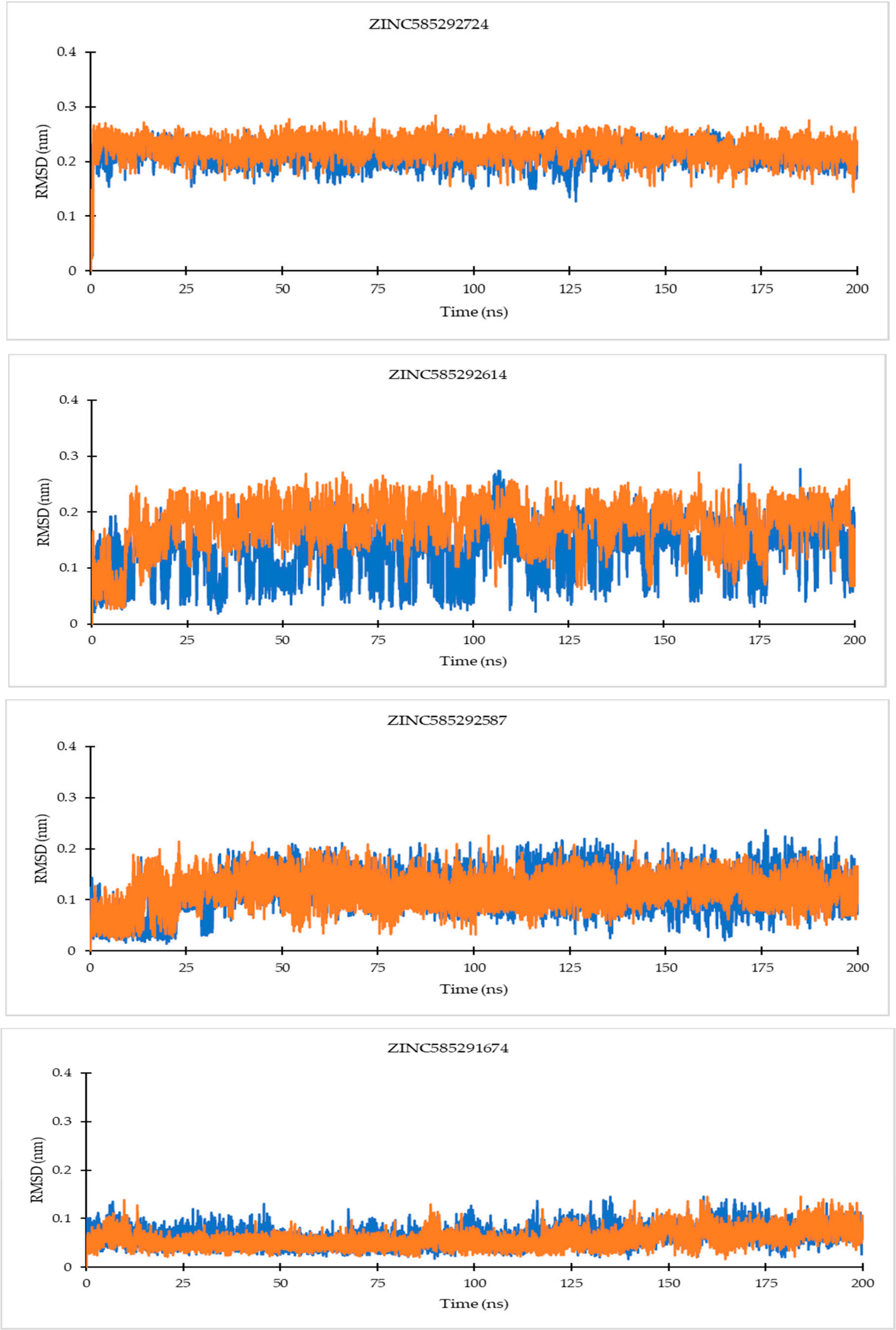

Figure S15: The ligand RMSD of the CDK6 complexes with ZINC585292724, ZINC585292614, ZINC585292587, and ZINC585291674. Color code: Blue: Replica 1; Orange: Replica 2.

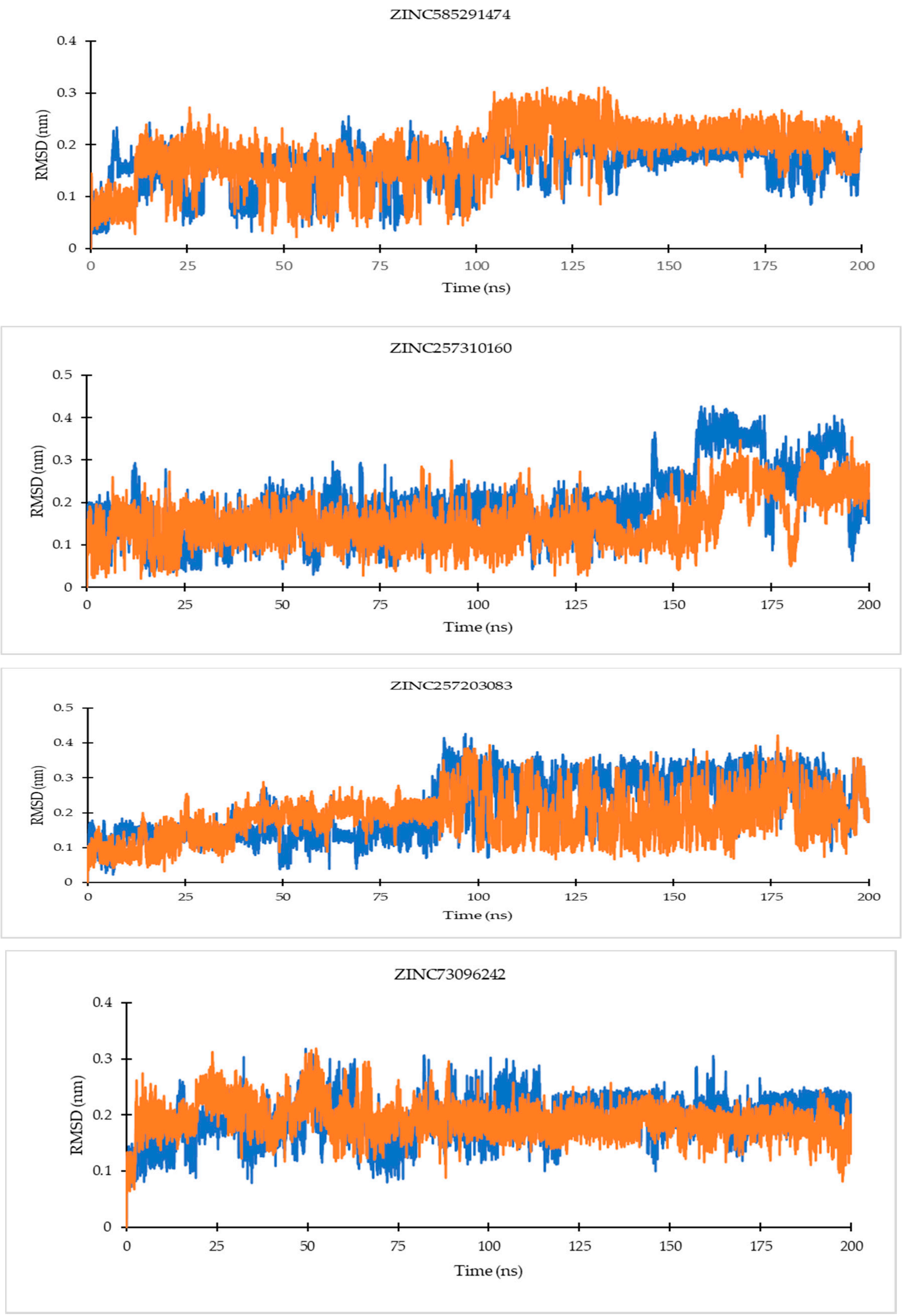

Figure S16: The ligand RMSD of the CDK6 complexes with ZINC585291474, ZINC257310160, ZINC257203083, and ZINC73096242. Color code: Blue: Replica 1; Orange: Replica 2.

Table S3. List of ligand-residue interaction throughout the simulations.

Ligands	Target			
	CDK4		CDK6	
	Ligand atom	Residue	Ligand atom	Residue
Palbociclib	N21	Val96(O)	N21	Val101(O)
	N23	His95(NE2)	N23	His100(NE2)
	O20	Lys35(NZ)	O20	LYS43(NZ)
	O1	Asp158(N)	O1	Asp163(N)
	N31	Asp99(OD2)	N31	Asp104(OD2)
ZINC585292724	N56	Val96(O)	N56	Val101(O)
	N58	His95(NE2)	N56	H100(ND1)
	O61	Lys35(NZ)	O61	LYS43(NZ)
	O61	Asp158(N)	O61	Asp163(N)
	N53	Asp99(O D2)	N56	Asp104(OD2)
ZINC585292614	O66	Val96(N)	N60	Val101(O)
	O66	His95(NE2)	N60	H100(ND1)
	N62	Lys35(NZ)	N64	LYS43(NZ)
	N64	Asp158(N)	N62	Asp163(N)
	O66	Asp99(N)	N59	Asp104(N)
ZINC585292587	O52	Val96(N)	N46	Val101(O)
	O52	His95(NE2)	N46	H100(ND1)
	N51	Lys35(NZ)	O52	LYS43(NZ)
	N46	Asp158(OD2)	N49	Asp163(N)
	N50	Asp99(N)	N44	Asp104(N)
ZINC585291674	N59	Val95(O)	N59	Val101(O)
	N62	His95(NE2)	O62	His100(NE2)
	O64	Lys35(NZ)	O64	LYS43(NZ)
	O64	Asp158(N)	O64	Asp163(N)
	N59	Asp99(OD1)	O61	Asp104(N)
ZINC585291474	N51	Val96(O)	N51	Val101(O)
	N54	His95(NE2)	N51	H100(ND1)
	O56	Lys35(NZ)	N49	LYS43(NZ)
	O56	Asp158(N)	N49	Asp163(N)
	N51	Asp99(OD2)	N53	Asp104(OD2)
ZINC257310160	O57	Val96(N)	N61	Val101(O)
	O57	His95(NE2)	N61	H100(ND1)
	O56	Lys35(NZ)	O57	LYS43(NZ)
	N61	Asp158(OD1)	N59	Asp163(N)
	N59	Asp99(OD1)	N54	Asp104(N)
ZINC257203083	N58	Val96(O)	N58	Val101(O)
	N60	His95(NE2)	N58	H100(ND1)
	O55	Lys35(NZ)	O55	LYS43(NZ)
	O55	Asp158(N)	N58	A163(OD1)
	N60	Asp99(N)	N59	Asp104(OD2)
ZINC73096242	N60	Val96(O)	N60	Val101(O)
	O56	His95(NE2)	N60	H100(ND1)
	O58	Lis35(NZ)	O58	LYS43(NZ)
	O58	Asp158(N)	N60	A163(OD2)
	O57	Asp99(N)	N58	Asp104(OD2)

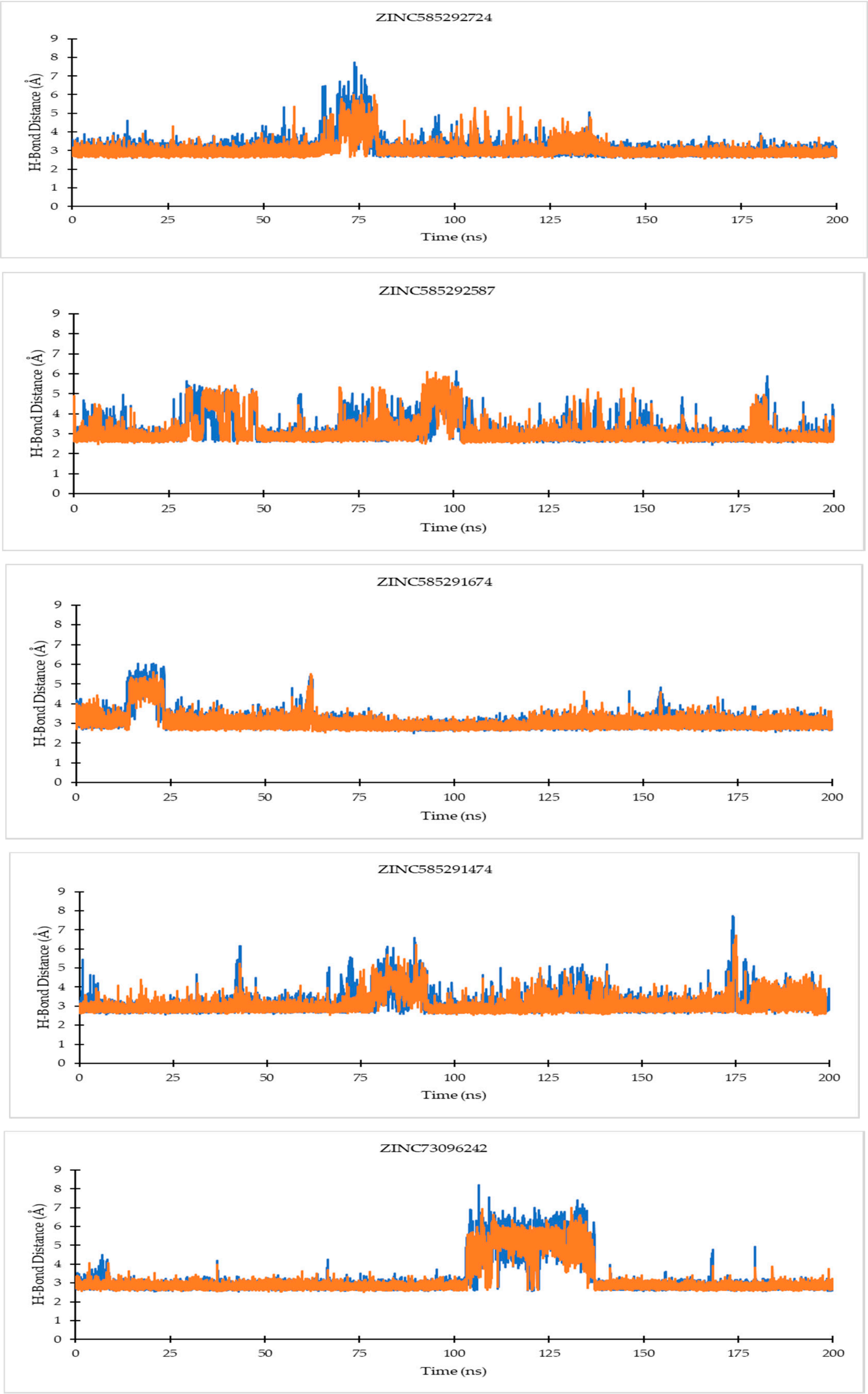

Figure S17. Hydrogen bond distances between ligand and Valine96 residue of CDK4 during the MD simulation. Color code: Blue: Replica 1; Orange: Replica 2.

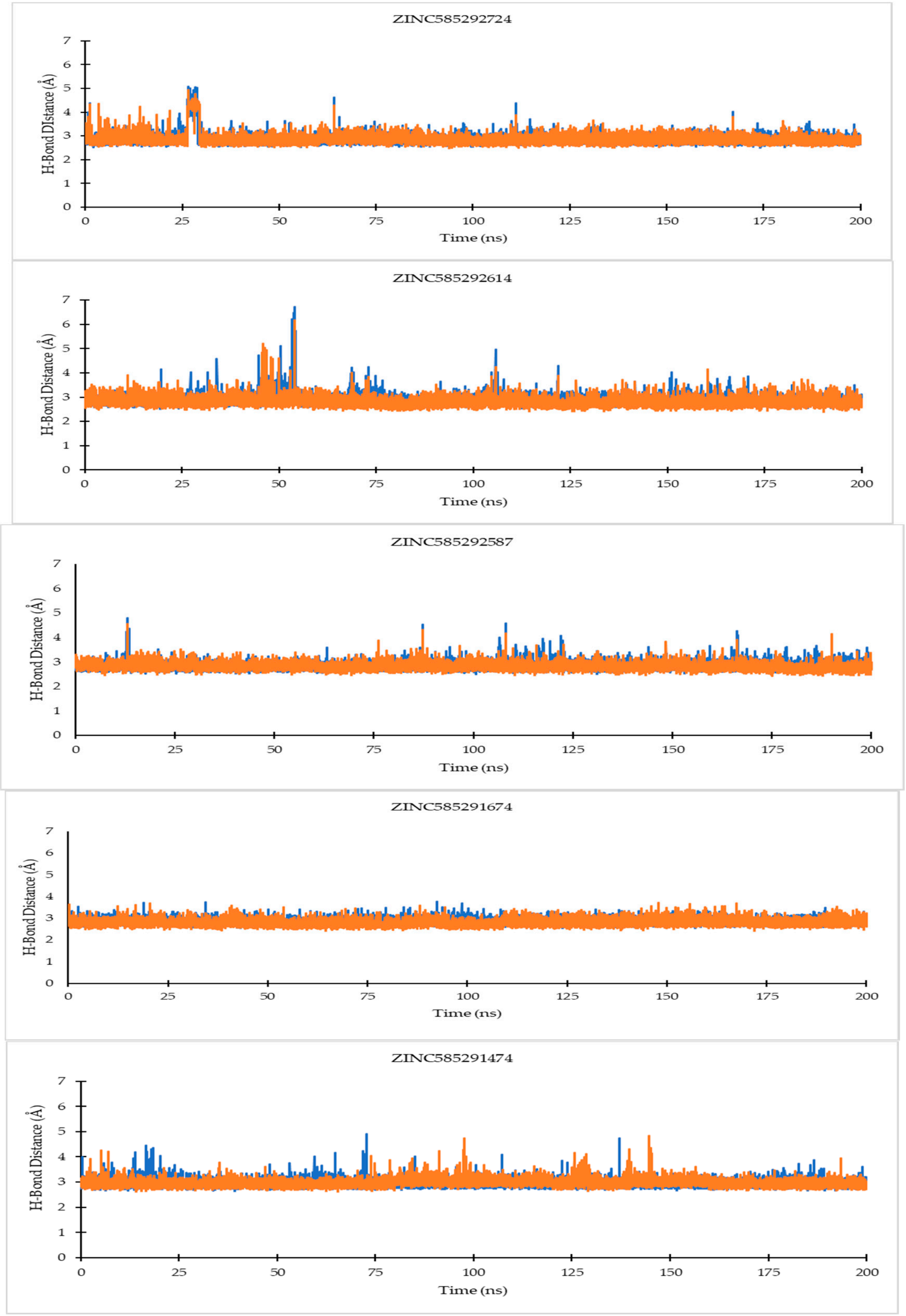

Figure S18. Hydrogen bond distances between ligand and Valine101 residue of CDK6 during the MD simulation. Color code: Blue: Replica 1; Orange: Replica 2.
